# VOC biomarker-based wearable gas sensors for real-time *in situ* monitoring of plant stress and health: applications and technical barriers

**DOI:** 10.3389/fpls.2026.1909243

**Published:** 2026-08-10

**Authors:** Shizhan Han, Pei Li, Xia Sun, Fei Sun

**Affiliations:** 1 School of Agricultural Engineering and Food Science, Shandong University of Technology, Zibo, China; 2 Institute of Modern Agricultural Equipment, Shandong University of Technology, Zibo, China; 3 Shandong Jiashibo Foods Co., Ltd, Weifang, China

**Keywords:** biotic and abiotic stress, *in-situ* detection, plant health monitoring, volatile organic compounds, wearable gas sensors

## Abstract

As an emerging *in situ* monitoring technique, wearable gas sensors provide a non-invasive and real-time monitoring tool for analyzing plant health status. When plants are subjected to biotic and abiotic stresses, they release characteristic volatile organic compounds (VOCs), which can serve as key biomarkers for early diagnosis. This paper systematically reviews the latest research progress of wearable gas sensors in the *in-situ* monitoring of plant health. Firstly, it systematically discusses the structural composition, key properties and major sensing mechanisms of the sensors. Subsequently, it outlines the applications of wearable gas sensors in monitoring plant growth and stress status, and analyzes in detail the design concepts and performance indicators in typical application cases. Finally, it thoroughly explores the core challenges currently faced by this technology and its future development directions. By bridging the application gap between material sensing and agricultural production, this review provides theoretical support and practical guidance for the construction of a precise prevention and control system for smart agriculture.

## 1 Introduction

Food security and ecological sustainability are central issues of global concern. As the primary producers in the Earth’s ecosystem, the health of plants directly impacts agricultural production, ecological balance, and human food security. During their growth, plants continuously release various volatile organic compounds (VOCs) into the atmosphere. These compounds are not only byproducts of plant metabolism but also serve as important signaling molecules in the interaction between plants and their environment. Decades of research have conclusively demonstrated that the types, concentrations, and release dynamics of plant VOCs are highly correlated with internal physiological and metabolic processes as well as various stress responses (Niederbacher et al., 2015). By monitoring these VOCs, it is possible to non-invasively obtain information on the physiological state of plants, thereby providing critical evidence for early diagnosis and targeted interventions of diseases. The stresses faced by plants can be divided into two main categories: biotic and abiotic stresses. Biotic stresses primarily stem from attacks by biological factors such as pathogenic microorganisms (e.g., fungi and bacteria), pests, and weeds. These factors often affect plant health by directly damaging plant tissues or inducing immune responses (Gimenez et al., 2018). Abiotic stresses include environmental factors such as drought, high salinity, extreme temperatures, heavy metal contamination, and nutrient imbalances. These factors usually do not exist in isolation but are interrelated and act synergistically or antagonistically, further amplifying their negative impacts on plants (Zhang et al., 2023).

During different stages of growth and development, plants release VOCs and metabolic gases such as ethylene, ammonia, and alcohols and aldehydes through organs such as leaves and flowers, forming a gas fingerprint profile that is highly correlated with their growth status. Under stress conditions, plants activate a series of defense mechanisms, the most notable of which is a significant increase in VOCs emissions. These VOCs encompass a wide range of chemical classes, including terpenes (such as α-pinene and β-pinene), phenolic compounds, benzenes, nitrogen- and sulfur-containing compounds, and fatty acid derivatives (such as the green-leaf volatiles C6 aldehydes and alcohols, methyl jasmonate, and cis-jasmonate produced via the lipoxygenase pathway) (Tholl et al., 2021; Ramakrishnan and Vijayan, 2026).The release of these compounds not only aids in plant self-repair and signal transduction but may also serve as “airborne signals” that influence neighboring plants or attract natural enemies, thereby forming complex ecological networks. As a result, VOCs have emerged as one of the most promising biomarkers for monitoring plant health.

**Figure 1:**
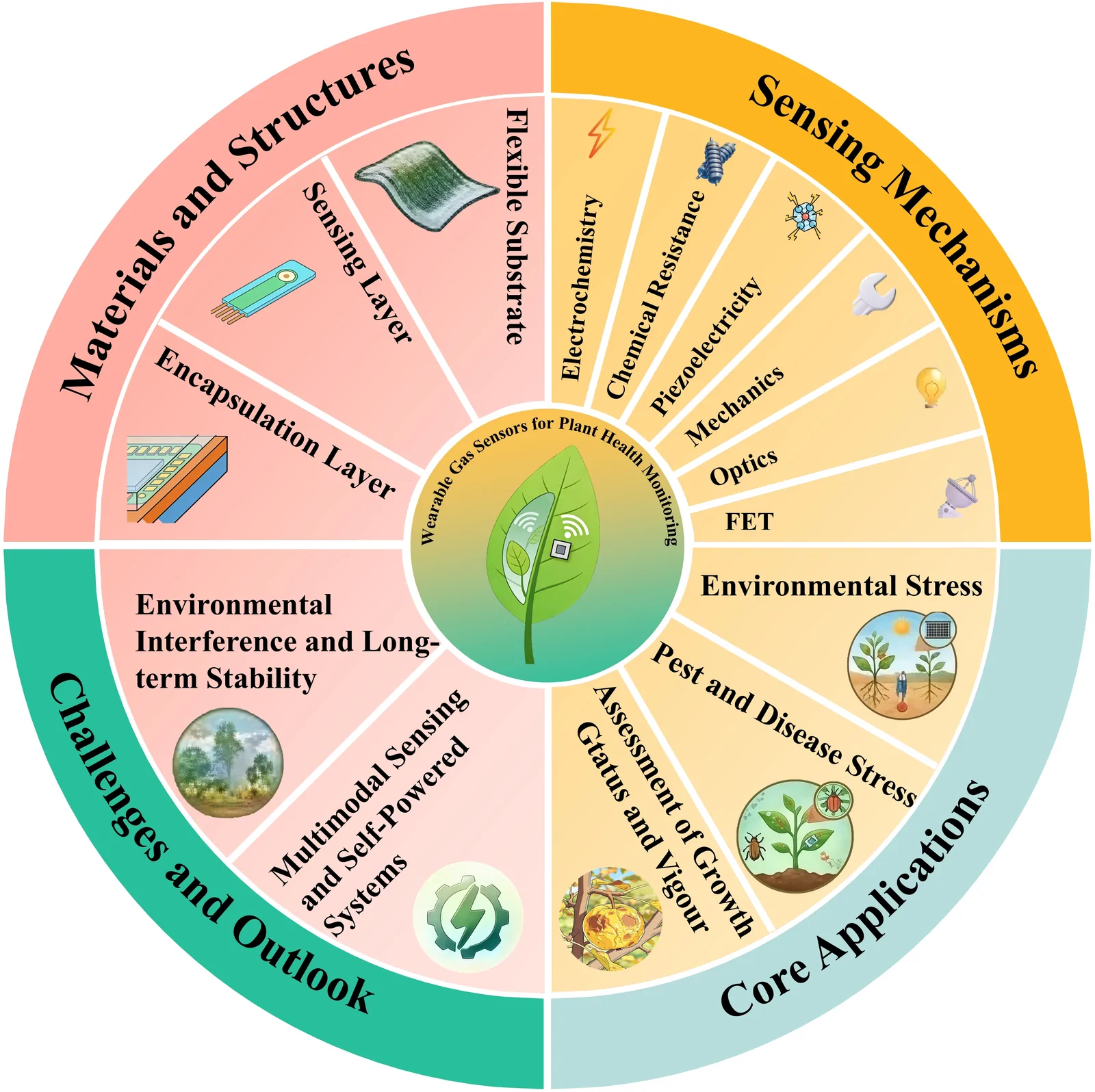
Schematic diagram of the main content of this review.

Traditional VOCs analysis techniques, such as gas chromatography-mass spectrometry (GC-MS) and liquid chromatography-mass spectrometry (LC-MS), have long dominated the field due to their extremely low detection limits (reaching the ppb or even ppt level) and exceptional separation and identification capabilities (Chai et al., 2023; Chary and Fernandez-Alba, 2012; Cheema et al., 2021). These technologies perform exceptionally well in laboratory settings, capable of accurately identifying target compounds in complex mixtures. However, their inherent limitations are becoming increasingly apparent: the instruments are bulky, expensive, and complex to operate, requiring specialized technicians. Furthermore, samples must be collected in the field and transported to the laboratory for analysis, which not only causes delays but may also introduce errors due to sample volatilization or degradation. These drawbacks make it difficult to meet the demands of modern agriculture for real-time, on-site, and cost-effective monitoring. Consequently, over the past few decades, a variety of gas sensors with rigid mechanical structures have been developed using different sensing materials and methods (Pathak et al., 2023; Jalal et al., 2018; Epping and Koch, 2023), including catalytic combustion, electrochemical, thermal conductivity, infrared absorption, paramagnetic, solid-state electrolyte, and metal-oxide-semiconductor (MOS) sensors. However, traditional rigid-structure sensors typically require high-temperature operation, resulting in high power consumption. Furthermore, their rigid substrates limit their application on flexible surfaces, and prolonged contact with crop tissues and organs can cause biological rejection and organ damage, making it difficult to meet the demand for long-term, continuous monitoring of crop physiological information. Wearable gas sensors made from flexible materials have overcome the limitations of traditional monitoring technologies thanks to their unique advantages, such as good conformal adhesion to plant surfaces, lightweight design, low disturbance, *in-situ* application, and real-time continuous monitoring. They are gradually being applied in the field of plant health monitoring. Flexible sensors exhibit mechanical properties similar to those of crop tissues and can be directly attached to plant surfaces (leaves, stems, fruits, etc.), enabling real-time capture, accurate quantification and continuous recording of characteristic gas signals emitted by plants. Without damaging plant tissues, they allow full-process tracking of health status changes during plant growth, providing a novel technical approach for early warning of plant stress, early diagnosis of diseases, and regulation of growth rhythms. Given the advantages of wearable gas sensors, **Figure 1** outlines the design, fabrication and application scenarios of this sensor in smart agriculture. Although existing reviews have summarized various types of VOCs sensors, there is still a lack of comprehensive reviews covering the entire chain of wearable VOCs sensors from material design, sensing mechanisms and system integration to practical applications. This has, to a certain extent, restricted the industrialization and field promotion of this technology.

Therefore, this paper first systematically introduces the structural composition, key properties and main sensing mechanisms of the sensors. Subsequently, it thoroughly investigates the latest advances of wearable gas sensors for *in situ* monitoring of plant health, with a focus on their performance in three key application scenarios: (1) monitoring of growth environmental stresses (e.g., drought, salt stress, nutrient deficiency); (2) early diagnosis of pest and disease stresses; (3) evaluation of plant growth status and vitality. It analyzes in detail the sensor design concepts, performance indicators, monitoring results and their revelations regarding plant physiological mechanisms. Finally, it dissects the core challenges currently encountered and provides forward-looking prospects combined with technological development trends. This review aims to offer theoretical support and practical guidance for follow-up research in this field, promote the large-scale application of wearable gas sensors in smart agriculture, and contribute to the precise and sustainable development of agriculture.

## 2 The relationship between VOCs emission and plant health status

VOCs emitted by plants serve as a sophisticated chemical language reflecting their internal physiological metabolism, developmental stage transitions, and interactions with the external environment. Under healthy conditions, plants produce background-level VOCs through basic metabolism, such as methanol, ethylene, and small amounts of terpenoids. However, upon exposure to biotic or abiotic stresses, their VOCs emission profiles undergo significant alterations: not only does the total quantity fluctuate, but the species, concentration ratios, and emission timing of specific compounds are also finely regulated. These characteristic gaseous molecules act as both internal signaling carriers and distress signals sent to the surrounding environment, forming the core biomarkers for plant health monitoring. A thorough understanding of the correlation between specific VOCs and plant physiological status is a critical prerequisite for moving from signal sensing to stress interpretation. This section systematically summarizes the emission patterns of typical VOCs and the specific physiological or stress conditions they indicate, providing a physiological basis for the targeted design and application of subsequent wearable sensors.

### 2.1 Biomarkers of basic metabolism and growth development

Normal physiological activities in plants are accompanied by specific emissions of VOCs, among which methanol serves as an important biomarker for basic metabolism and growth and development. Methanol emission mainly originates from the demethylation of cell wall pectin, catalyzed by pectin methylesterase, which is directly related to cell elongation and cell wall remodeling. Pectin methylesterase activity is regulated by plant developmental stage and hormone signals (such as auxin and abscisic acid), playing a key role in cell wall loosening and expansion. This explains why young tissues and plants at rapid growth stages exhibit higher baseline methanol emission levels (Ibrahim et al., 2022; Sangmen et al., 2026). Consequently, methanol is released at relatively high background levels in rapidly growing crops or young tissues, reflecting vigorous metabolic activity. Ibrahim et al. (2022) confirmed using *in situ* monitoring with wearable sensors that young maize plants at the V8 growth stage emitted significantly more methanol than those at the V12 stage, a difference indicative of higher metabolic activity during early vegetative growth. Conversely, methanol emission profiles change markedly under growth arrest or nutrient stress. Quantitative evidence was provided by Sangmen et al. (2026), who reported that the average methanol emission concentration in cotton plants under nitrogen deficiency reached 17.65 ppm, compared with only 3.81 ppm under sufficient nitrogen supply, demonstrating that nitrogen availability profoundly affects methanol production and release. It should be noted that the release of methanol is not unique to nitrogen deficiency—mechanical damage, pathogen infection and sudden changes in ambient temperature can also induce a significant increase in its release; therefore, when using it as a diagnostic indicator of nitrogen deficiency, a comprehensive assessment must be made in conjunction with other supporting parameters. Besides methanol, other VOCs are also closely coupled with specific developmental stages. As an endogenous hormone, ethylene is released in large quantities during fruit ripening and organ senescence, driving color transformation and nutrient redistribution. Benzene-ring and terpenoid compounds emitted by floral organs form species-specific “floral scent fingerprints”, whose emission rhythms are precisely synchronized with pollination windows, making them potential indicators for evaluating reproductive growth vigor. The dynamic emission of these VOCs collectively delineates the physiological status of plants across different life cycles.

### 2.2 Biomarkers for abiotic stress responses

When plants are exposed to abiotic stresses such as drought, salinity, or nutritional imbalance, their internal signaling networks rapidly reshape the synthesis and release of VOCs, providing specific chemical fingerprints for the early diagnosis of stresses. Under drought stress, although reduced stomatal conductance limits gas exchange, the upregulated expression of terpene synthase genes alters emission profiles. The molecular mechanism primarily involves the activation of the abscisic acid (ABA) signaling pathway—drought stress induces substantial ABA accumulation, which in turn regulates the transcriptional levels of terpene synthase (TPS) gene families, promoting the synthesis and release of monoterpenes such as α-pinene (Zheng et al., 2023a). Zheng et al. (2023b) monitored Chinese juniper and found that 40 consecutive days of drought stress increased the sensor resistance by approximately 2% due to elevated α-pinene release, indicating that α-pinene can serve as a stable indicator of drought stress.

However, the release of α-pinene is also induced by insect feeding and mechanical damage, and its emission levels are closely related to plant species, leaf age, and temperature and humidity conditions; it is therefore not a specific marker of drought stress. Salt stress mainly activates the ethylene synthesis pathway through ion toxicity and osmotic stress. Studies by Sholehah et al. (2021) and Yan et al. (2023) confirmed that sensors based on ZnO-Ag or SWCNT-PdNP composite materials can sensitively detect ethylene at ppm to ppb levels, whose concentration changes can be used as a probe for the early warning of salt damage. As a key stress hormone, ethylene is released in response to a variety of stresses (including drought, waterlogging, disease and mechanical damage); consequently, its use in diagnosing salt stress must also be cross-validated in conjunction with other physiological indicators or multi-sensor arrays. Regarding nutritional stress, research by Sangmen et al. (2026) provided direct evidence for the quantitative relationship between nitrogen deficiency and VOCs emissions: the average methanol emission concentration of nitrogen-deficient cotton plants (17.65 ppm) was 4.6 times that of plants under sufficient nitrogen (3.81 ppm), and the average α-pinene emission (1217.49 ppb) was 2.3 times that of nitrogen-sufficient plants (529.82 ppb).This confirms that nutrient deficiency induces reproducible and quantitative changes in specific VOCs. These differential response patterns of VOCs form the chemical basis for discriminating stress types and intensities in precision agriculture.

### 2.3 Biomarkers for biotic stress defense

Plants’ defense responses against pests and diseases are accompanied by dramatic remodeling of VOCs emission profiles, in which green leaf volatiles, terpenoids, and aromatic compounds play central roles. Green leaf VOCs represented by (E)-2-hexenal are immediate products of the film lipid peroxidation pathway when plant tissues suffer mechanical damage or pathogen invasion. Their rapid release allows them to act as the “first alarm” for insect feeding and disease infection. Li Z. et al. (2021) developed a chemiresistive sensor array based on reduced graphene oxide (rGO) and functionalized gold nanoparticles, which detected significant changes in VOCs responses as early as 4 days after inoculation with Phytophthora infestans (late blight), prior to the appearance of visible symptoms. Another study using a smartphone-based colorimetric sensor array (Li Z. et al., 2019) further lowered the detection limit of (E)-2-hexenal to 0.4 ppm, enabling discrimination between late blight and healthy controls at 2 days post-inoculation, as well as differentiation between early blight and Septoria leaf spot, with a blind test accuracy of over 95%. Terpenoids are abundantly synthesized during the later stages of defense. Weerakoon et al. (2012) constructed sensor arrays using polymer/carbon composites, polythiophenes, and other materials, successfully detecting and distinguishing six insect-induced terpenoids including γ-terpinene, α-pinene, limonene, and farnesene. Characteristic fingerprints generated via principal component analysis provided a technical route for early warning of insect pests. In addition, methyl salicylate and methyl jasmonate, as volatile forms of plant immune hormones, signal the establishment of systemic acquired resistance and can transmit warning signals between plants, priming neighboring healthy plants for defense in advance (Ramakrishnan and Vijayan, 2026). Dynamic monitoring of these defense-related VOCs represents a key strategy for achieving early, non-destructive diagnosis of pests and diseases and evaluation of population resistance.

It is worth noting that, although there are statistically significant correlations between the aforementioned VOCs and specific stresses, the vast majority of VOCs are not specific biomarkers unique to any single stress. Methanol is released in large quantities under nitrogen-deficient conditions and is also significantly up-regulated in response to mechanical damage and pathogen infection (Ibrahim et al., 2022; Sangmen et al., 2026); α-pinene exhibits increased emissions under conditions of drought, insect damage and mechanical damage (Zheng et al., 2023b; Weerakoon et al., 2012); (E)-2-hexenal is rapidly released during both pathogen infection and mechanical damage (Li Z. et al., 2021; Li Z. et al., 2019); ethylene, as a key stress hormone, exhibits a broad-spectrum response to drought, salinity, disease and mechanical damage (Sholehah et al., 2021; Yan et al., 2023, 2024; Wang et al., 2013). Consequently, relying solely on a single VOC to identify the type of stress carries a significant risk of misclassification. Previous studies have confirmed that by constructing cross-responsive sensor arrays and combining them with principal component analysis or machine learning algorithms, it is possible to achieve accurate classification of complex VOC mixtures and identification of stress types (Li Z. et al., 2019; Weerakoon et al., 2012). Song et al. (2026) recently reported a tunable, highly sensitive integrated chemical gas sensing system based on functionalized carbon nanotubes; through an arrayed design combined with pattern recognition algorithms, it achieved precise discrimination between multiple chemical gases. This strategy provides an important technical reference for the construction of plant VOC fingerprint profiles. Taking the above evidence into account, accurate plant health diagnosis must rely on the combined application of VOC fingerprints, sensor arrays and machine learning/pattern recognition algorithms, rather than on the detection of a single analyte. This understanding also serves as the fundamental logical premise for the selection of various sensor designs and data analysis strategies in subsequent chapters.

**Figure 2:**
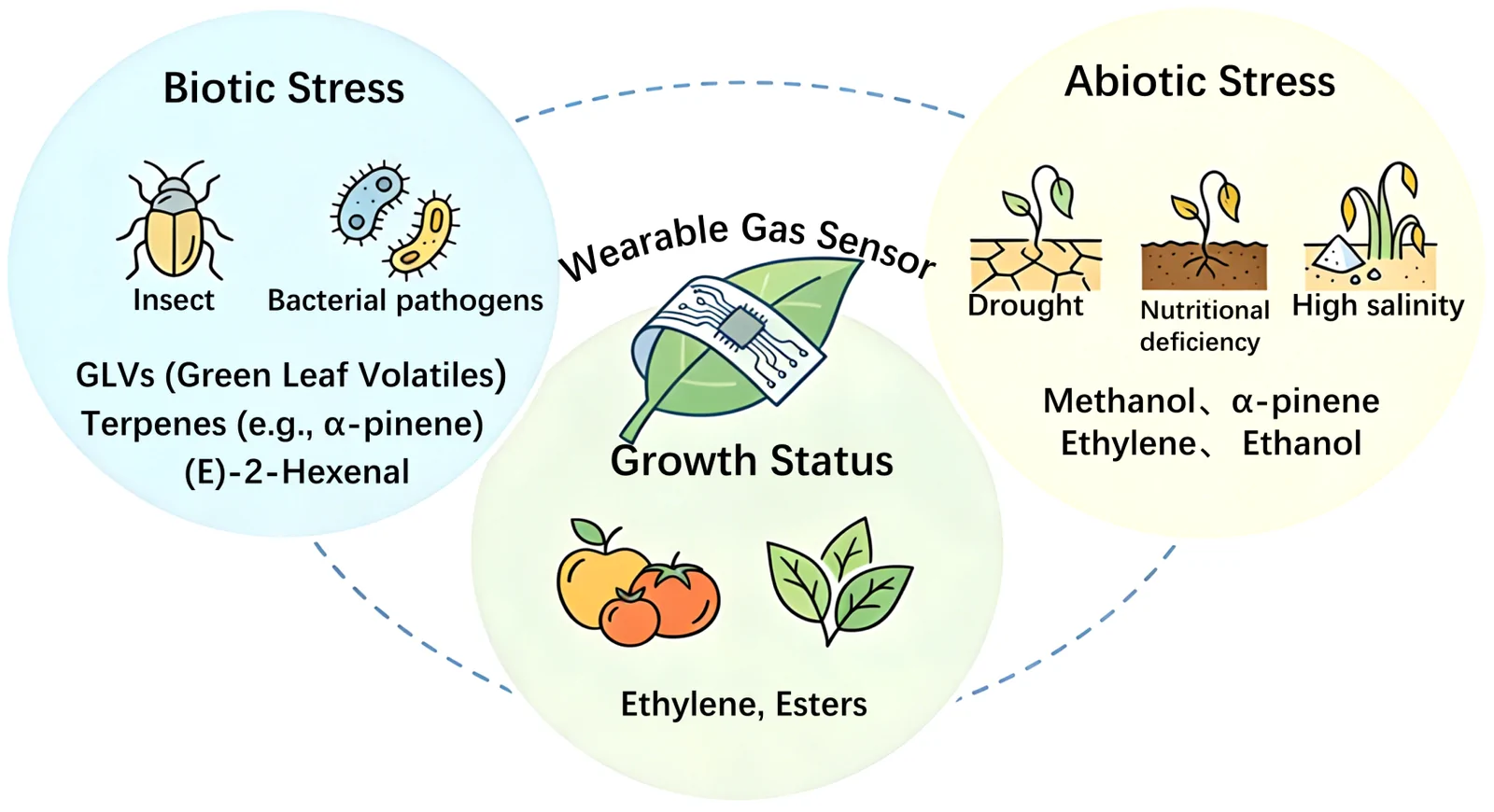
Stress and its classification.

In summary, the emission of plant VOCs represents a chemical phenotype resulting from their interaction with complex environments. Establishing a quantitative mapping relationship between the concentration thresholds, release kinetics, and combinatorial patterns of specific VOCs and plant physiological status, stress type, and stress intensity constitutes the fundamental logic guiding material selection, device design, and data interpretation for wearable gas sensors. The subsequent analysis of the applications of various sensor types in this chapter represents concrete technical practices based on the above-mentioned VOCs - status correlations. **Figure 2** clearly illustrates the various categories of plant stress and growth metabolism, helping us gain an intuitive understanding of the complexity of plant growth.

To avoid oversimplifying the correlation between VOC emissions and stress types, and considering that plant VOC biosynthesis is strongly modulated by environmental factors such as temperature through cell wall metabolism and secondary metabolic pathways (Resente and Crivellaro, 2025), we summarize the typical VOC biomarkers, corresponding stress types, plant species, detection tissues, concentration ranges, validation methods, and major confounding factors in **Table 1**.

**Table 1:** Summary of the relationship between the release of volatile organic compounds (VOCs) and plant stress.

Types of VOCs	Main associated stresses	Plant species (examples)	Tissues analyzed	Concentration range	Validation methods	Interfering factors/limitations
Methanol	Nitrogen deficiency stress, mechanical damage, insect damage	Cotton, maize	Leaves	3.81–17.65 ppm	Wearable sensors vs GC-MS	Wearable sensors vs GC-MS Significantly influenced by developmental stage (young leaves release higher levels than mature leaves) and temperature and humidity; not a marker specific to nitrogen deficiency
α-Pinene	Drought stress, mechanical damage, insect damage	Thuja, cotton, various crops	Leaves, stems	0.5–40 ppm (detection range); 529.82–1217.49 ppb (actual emissions)	Chemoresistive sensor, GC-MS	Significant differences in release characteristics between conifers and broad-leaved trees; overlap between drought and insect damage signals
(E)-2-hexenal	Diseases (late blight, etc.), mechanical damage	Tomato, potato	Leaves	LOD 0.4–0.61 ppm	Colorimetric sensor, fluorescence sensor, GC-MS	Release kinetics differ between mechanical damage and disease-induced release, but it is difficult to distinguish between them based on a single compound alone
Ethylene	Salt stress, fruit ripening, senescence, various stresses	Rice, Arabidopsis, tomato, banana	Leaves, fruit	31 ppb–30 ppm	Chemical resistance sensors, QCM, GC-MS	A broad-spectrum stress hormone that responds to a variety of stressors but lacks specificity; must be used in conjunction with other indicators
γ-terpinene, limonene and other terpenes	insect damage	A variety of crops	Leaves	100 ppm (as detected by the sensor)	Polymer sensor arrays, GC-MS, principal component analysis	Terpenes are structurally similar and can only be distinguished using a sensor array combined with pattern recognition
Volatile compounds from green leaves (C6 aldehydes, alcohols, etc.)	Diseases, mechanical damage, insect damage	Tomato	Leaves	ppb–ppm range	Chemical resistance sensor array, GC-MS	The release patterns of mechanical damage and disease-induced damage differ, but it is difficult for a single sensor to distinguish between them

## 3 Structure, characteristics, and sensing mechanisms of wearable gas sensors

### 3.1 Sensor structure and key components

Wearable gas sensors typically employ a classic three-layer sandwich architecture: a flexible or stretchable substrate, a sensing layer (or electrode), and a protective encapsulation layer (Kim et al., 2018; Amjadi et al., 2014; Roh et al., 2015). The sensing active layer is positioned between the substrate and the encapsulation layer, forming a stable functional unit (Lana et al., 2019). Flexible substrates and sensing materials undergo a series of processing steps and are combined with encapsulation to form the sensor’s main structure. By modifying and integrating conductive circuits, laptops or mobile phones for information collection can eventually be applied to plant health monitoring.

#### 3.1.1 Substrate and packaging materials

The substrate material is key to determining the sensor’s flexibility, stretchability, and biocompatibility (Cao et al., 2008; Zhang et al., 2012; Wang et al., 2019; Lu et al., 2022). Although traditional rigid substrate (such as glass (Hsueh et al., 2011; Sharma et al., 2020), silicon dioxide/silicon (Hassan et al., 2013), aluminum oxide (Patel et al., 2003), and indium tin oxide (ITO)-coated glass (Wang et al., 2007)) widely are used in flat devices, these materials struggle to meet the bending, stretching, and twisting requirements of wearable applications. As a result, researchers have turned to flexible polymeric materials, including elastomers such as polyethylene terephthalate (PET), polyimide (PI), polydimethylsiloxane (PDMS), and polyvinyl alcohol (PVA) (Yeasmin et al., 2023; Khan et al., 2021a; Bukhamsin et al., 2022; Khan et al., 2021b; Huang et al., 2020). Thanks to their excellent flexibility, mechanical stability, and conformability, these materials have become the preferred choice for wearable gas sensor substrates. For example, Cai et al. (2023) developed a wearable NH_3_ gas sensor based on a polyaniline/cobalt porphyrin (PANI/CoTPP) composite sensing film. The sensor uses a flexible ITO-PET substrate, with the composite sensitive film deposited onto the substrate surface via a one-step self-assembled electrodeposition method. Through characterization analysis and gas performance testing, it achieves highly sensitive detection of low-concentration NH_3_, with a response value of 12.5% at 1 ppm NH_3_ and a detection limit as low as 0.15 ppm. The response/recovery times are 18 s and 25 s, respectively. Furthermore, the response retention rate remains above 90% after 500 bending cycles with a bending radius of 5mm. The incorporation of the PET substrate effectively endows the sensor with excellent mechanical stability, making it suitable for wearable applications. Similarly, Maity et al. (2018) developed a wearable ethanol gas sensor based on a PDMS/PVA composite substrate, using a PDMS elastic film as a flexible support, with PVA serving as the dispersion and encapsulation layer for multi-walled carbon nanotubes (MWCNTs). By applying a PVA/MWCNTs composite solution to the fabric surface via dip-coating and encapsulating it with PDMS, the device achieved rapid, highly selective detection of ethanol in the range of 100–500 ppm, with a response value of 13 at 500 ppm and a detection limit of 9.17 ppm. The response/recovery times are 24 ± 2 s and 29 ± 3 s, respectively. The synergistic effect of PDMS and PVA not only enhances the sensor’s tensile properties and conformability but also ensures that baseline resistance fluctuations remain below 5% after 1,000 cycles of 60° bending. This makes it suitable for real-time monitoring of ethanol vapor in human breath and the environment, further validating the application value and advantages of flexible elastomer substrates in the field of wearable gas sensing. These materials feature low Young’s modulus, high tensile strength, and good optical transparency, making them suitable for adhesion to plant surfaces. Furthermore, hydrogels, as three-dimensional network polymers with high water content, possess excellent flexibility and biocompatibility and have already been applied in plant sensors (Ju et al., 2021; Karimzadeh et al., 2022; Lin et al., 2022; Li Z. et al., 2021). Wu et al. (2019) developed a highly flexible gas sensor based on an ion-conductive dual-network hydrogel. This sensor uses a polyacrylamide/κ-carrageenan hydrogel as its matrix; its three-dimensional porous network and high water content provide pathways for gas transport and ion conduction. Signal output of the sensor is achieved through changes in electrical conductivity caused by the interaction between gas molecules and the hydrogel’s functional groups, demonstrating high sensitivity and low detection limits for gases such as NO_2_ and NH_3_. It also exhibits excellent tensile strength and deformation stability, allowing it to be applied to plant surfaces for wearable gas monitoring, thereby providing a new flexible sensing solution for detecting environmental and stress-related gases in plants. In recent years, environmental sustainability and biodegradability have become new directions in materials design. Natural materials such as cellulose, lignin (Wang et al., 2023), pollen (Hwang et al., 2022), and leaves (Yun et al., 2015) have been used as green substrates. This not only reduce the environmental impact of electronic waste but also provide a feasible pathway toward “green electronics” in the future (Gomes et al., 2023; Teixeira et al., 2023). Barandun et al. (2019) fabricated near-zero-cost wearable gas sensors by utilizing cellulose paper, whose renewable and biodegradable properties provide a low-cost, sustainable solution for green wearable sensing. The development of these materials reflects the transition of wearable sensors from laboratory prototypes to sustainable agricultural applications. The pursuit of sustainable and biodegradable substrate materials has further expanded the material library for plant-wearable sensors. Recent advances in cellulose-based materials have demonstrated remarkable potential as green substrates for flexible electronics. For instance, bamboo cellulose-derived carbon nanomaterials (C-BCN) have been incorporated into polyacrylamide hydrogels to achieve ultra-robust mechanical properties (tensile strength: 363 kPa, toughness: 3.04 MJ/m³) with enhanced fracture resistance, offering a renewable alternative to petroleum-based substrates (Duan et al., 2025). Similarly, catalyst-free cellulose ionogels have achieved tensile strengths up to 11 MPa, a tenfold improvement over conventional cellulose gels, while maintaining ionic conductivity (29.1 mS/cm) and environmental sustainability (Yang et al., 2025). These cellulose-based platforms exhibit inherent biocompatibility, biodegradability, and abundant surface functional groups for sensor integration, positioning them as promising candidates for next-generation, eco-friendly plant-wearable sensing systems.

The primary purpose of the encapsulation layer is to enhance the sensor’s mechanical robustness, environmental tolerance, and service life. Although encapsulation may slightly increase response/recovery times or reduce peak sensitivity, it significantly expands the linear dynamic range and increases the tensile limit. For example, Nguyen et al. (2021) encapsulated the flexible sensor made of pleated metal film, expanding the linear dynamic range by approximately 50% and increasing the tensile limit to 260%, thus significantly enhancing the mechanical robustness and environmental stability of the devices. Additionally, in some designs, the encapsulation layer can be omitted, allowing the sensor to function effectively using only the substrate and active layer (Zhou et al., 2021; Zeng et al., 2019). Li H. et al. (2021) developed an unencapsulated paper-cut flexible substrate sensor, integrated with a rGO array. Without the need for encapsulation, it can be directly attached to tomato leaves to enable highly sensitive, real-time monitoring of VOCs associated with 13 types of plant stress, providing early warning of late blight four days in advance.

#### 3.1.2 Sensitive layer materials

**Figure 3:**
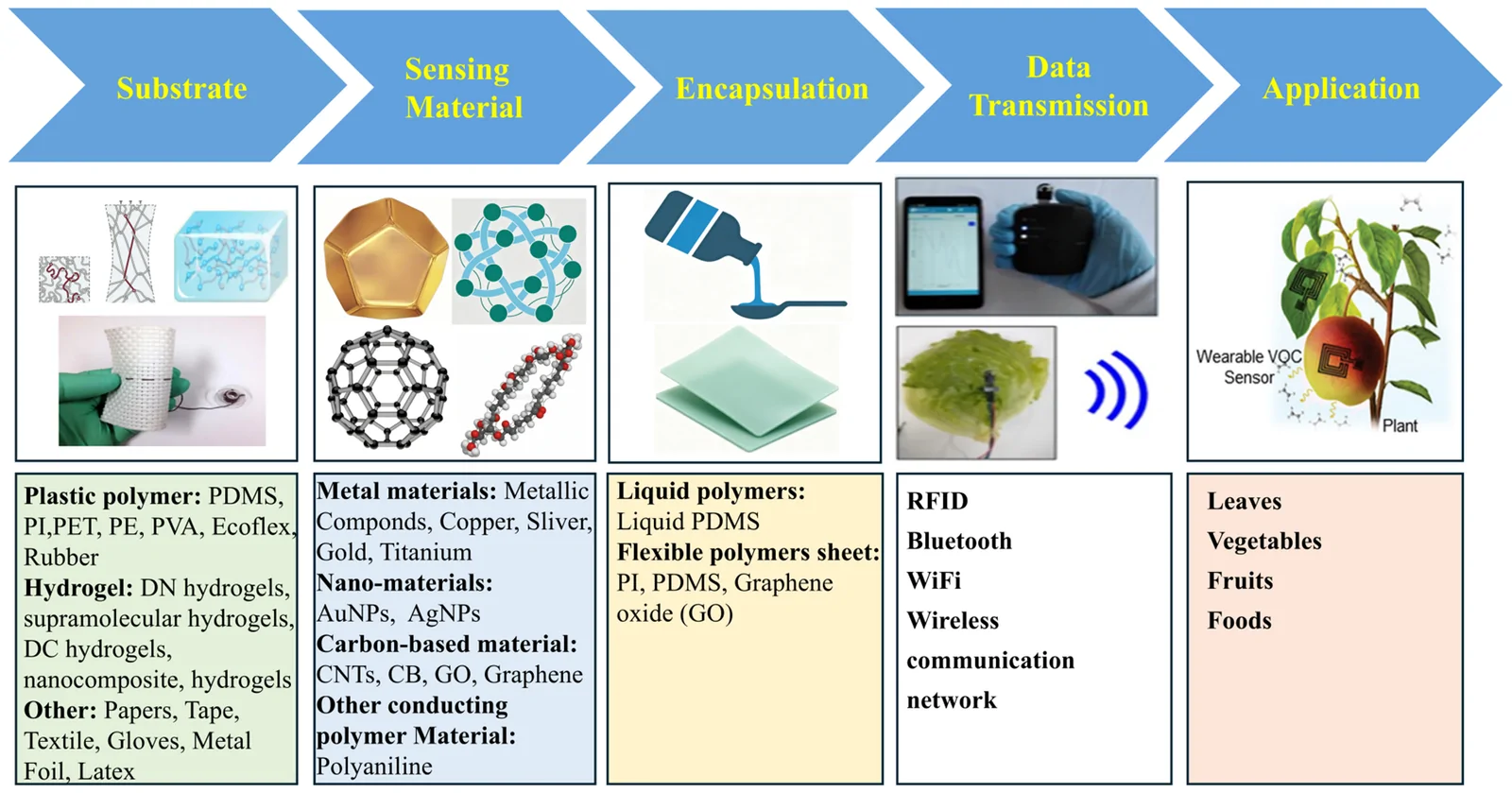
The basic components of wearable gas sensors.

The sensing element is the “brain” of the sensor. The choice of sensing element or electrode depends on the specific testing and monitoring requirements for plant health. Numerous studies have reported on the various sensing materials used in wearable plant sensors (**Figure 3**). Common active materials can be broadly categorized into several groups. Carbon-based materials include graphene, reduced graphene oxide (rGO), graphene oxide (GO), carbon black (CB), carbon spheres (CSS) (Raymundo-Pereira et al., 2021), carbon nanofibers (CNFs, herein referring to carbon nanofibers to distinguish them from cellulose nanofibers) (Lawaniya et al., 2023), carbon nanotubes (CNTs) (Basu and Bisker, 2025), carbon nanospheres (Paschoalin et al., 2022), and Printex carbon nanospheres (PCNB) (Paschoalin et al., 2022). Carbon-based nanomaterials exhibit outstanding mechanical and electrical properties, making them widely adopted in flexible gas sensors. For example, rGO functionalized with gold nanoparticles have been employed in chemiresistive sensor arrays for real-time monitoring of 13 types of plant VOCs (Li Z. et al., 2021). Metals and metal nanoparticles, such as gold (Au), titanium (Ti), silver (Ag), copper (Cu), platinum (Pt), and palladium (Pd), are commonly used as electrode materials or catalytic sensitizers (Raman and Arunagirinathan, 2022). Gold nanoparticles (AuNPs) have been widely utilized as functionalization agents to enhance the sensitivity and selectivity of carbon-based gas sensors (Li S. et al., 2019). For instance, Au interdigitated electrodes coated with PEVA/MWCNT composites have been developed for wireless α-pinene sensing (Zheng et al., 2023b). It should be noted that, due to their high Young’s modulus, metallic materials are prone to fracture when subjected to even low tensile strains, resulting in discontinuities in the film’s microstructure. In contrast, nanomaterials, with their superior mechanical and electrical properties, offer high spatio-temporal resolution for detecting water-soluble or volatile plant signaling molecules (Jung et al., 2019). Conductive polymers, such as polyaniline (PANI), polypyrrole (PPy), polythiophene, and their composites, have been extensively investigated for room-temperature gas sensing due to their tunable conductivity and reversible doping/dedoping properties (Xu et al., 2021). For example, PANI/CoTPP composites deposited on flexible ITO-PET substrates have shown high sensitivity to NH_3_ at room temperature (Cai et al., 2023). Metal-organic frameworks (MOFs) have recently emerged as promising sensing materials owing to their high porosity, large surface area, and tunable pore structures. Representative examples include UiO-66(Zr)-based composites loaded with colorimetric dyes for VOC detection (Wang et al., 2024b), and Cu-MOF-74 incorporated with SWCNTs and PdNPs for selective ethylene sensing (Yan et al., 2024). Biomaterials and bio-inspired materials, such as cellulose, chitosan, and agarose hydrogels, are gaining attention for their biodegradability, biocompatibility, and sustainability. For instance, agarose hydrogels loaded with aggregation-induced emission gold nanoclusters have been used for portable fluorescence detection of (E)-2-hexenal (Gan and Wang, 2024). Hybrid composites combining two or more of the above categories have shown synergistic effects that enhance sensing performance. These include Au@Ag core-shell nanowires with MWCNTs for multimodal VOC sensing (Lee et al., 2023), PtNPs/ZnONRs@ZIF-8 nanocomposites for methanol detection (Sangmen et al., 2026), and PdNPs-SWCNTs@Cu-MOF-74 hybrids for ethylene sensing (Yan et al., 2024). Among these, carbon-based nanomaterials such as graphene, GO, CNTs, CB, and CNFs have found widespread application in novel electronic devices due to their outstanding mechanical and electrical properties (Kang et al., 2021). For example, Lee et al. (2023) developed a multimodal wearable sensor by constructing a three-dimensional hybrid active network using gold-coated silver nanowires (Au@AgNWs) and MWCNTs, realizing the simultaneous detection of plant stress-related VOCs, temperature, and humidity. Similarly, Li Z. et al. (2021) fabricated an 8-channel gas-sensing array using AuNPs functionalized with rGO as the active layer. It can be directly attached to leaves without encapsulation, enabling highly sensitive detection of 13 types of plant VOCs and early warning of plant diseases, thereby fully demonstrating the core application value of metal and carbon-based nanomaterials in wearable plant gas sensing. In addition, conductive polymer composites composed of a non-conductive matrix and a conductive or semiconducting dispersed phase are also commonly used in plant health monitoring (Ryuzaki et al., 2017; Wang et al., 2016). Armitage et al. (2020) systematically investigated the gas sensing performance of polypyrrole (PPy) permeation networks on flexible PET substrates. By leveraging the limited conductive pathways near the permeation threshold, the detection limit for ammonia was reduced to below 10 ppb, laying a theoretical foundation for the application of conductive polymer composites in flexible plant sensors. Advanced sensing materials, including nanomaterials, metals, and conductive fibers, play a crucial role in achieving high sensor sensitivity (manifested as low detection limits and wide linear ranges). These materials also enhance sensor stability and ensure a longer service life through their unique properties. For example, Kim et al. (2026) developed a wearable plant gas sensor modeled after the pad structure of amphibian toes. By physically embedding MWCNTs electrodes into a PDMS substrate, the sensor exhibits excellent mechanical robustness, the electrodes remained intact even after 10 washing cycles, and the sensor maintained stable detection performance under both convex and concave bending conditions. Zhuang et al. (2024) developed a flexible ammonia sensing system using a composite of PANI and MWCNTs. It has been clearly demonstrated that this sensor exhibits a long lifespan and maintains stable performance under varying humidity conditions and repeated bending cycles. Additionally, the use of a flexible substrate allows the sensor to conform to the surface of plants, facilitating on-site monitoring and ensuring high detection accuracy.

### 3.2 Primary sensing mechanism

The core of wearable gas sensors lies in converting the interaction between gas molecules and the material into measurable electrical or optical signals (Li et al., 2024). The following provides a detailed explanation of the main mechanisms.

#### 3.2.1 Electrochemical sensing mechanisms

Electrochemical sensors convert chemical information into changes in current, potential, or conductivity based on redox reactions that occur at the electrode-electrolyte interface involving the target gas (Zeng et al., 2016). Based on their operating principles, they can be classified into potential-type (based on changes in the potential difference according to the Nernst equation), conductivity-type (based on changes in conductivity), and amperometric-type (based on current response at a constant potential). Among these, amperometric sensors are the most widely used due to their high sensitivity, fast response, and excellent quantitative capabilities (Pereira et al., 2024; Wang J. et al., 2018). In the field of plant monitoring, amperometric electrochemical sensors have demonstrated significant potential for application. Ibrahim et al. (2022) further developed an amperometric gas sensor with a conductive polymer/platinum nanoparticle composite sensing layer by using a typical three-electrode system, which can be directly attached to the surface of leaves, and achieved high sensitivity detection of methanol gas on the leaves of corn plants at a constant potential of 0.47V (detection limit is 0.5 ppm, and response time is 10s). It is worth noting that, beyond the typical electroactive gases such as NO_2_ and NH_3_, the detection of weakly adsorbing and low-polarity molecules (e.g., ethylene) poses significant challenges for electrochemical sensing, often requiring catalyst-assisted reactions or interfacial functionalization strategies to achieve adequate sensitivity and selectivity. Electrode materials, electrolyte composition, and interface modification are key areas for improving selectivity and stability (Williams, 2020). For example, an enzyme-catalyzed electrochemical wearable gas sensor has been developed by Zhang et al. (2024a). This sensor utilizes alcohol dehydrogenase as a specific catalytic element, which is immobilized on the surface of an electrode modified with carbon-based nanomaterials. Employing a three-electrode system, the sensor generates a detectable current signal through the specific redox reaction between the enzyme and ethanol gas, achieving high sensitivity (26.27 μA mM^-1^ cm^-2^) and a low detection limit (0.16 μM) for ethanol gas. The sensor can be integrated onto flexible substrates to suit wearable applications, enabling real-time monitoring of ethanol gas in human breath or the environment. The study also confirms that electrode material modification and the design of the enzyme immobilization interface are key factors in enhancing the stability and selectivity of this wearable sensor.

#### 3.2.2 Mechanism of chemical resistance sensing

Chemoresistive sensors (also known as resistive or chemoresistive sensors) operate on the principle of changes in material resistance caused by gas adsorption (Ghorbani and Taghipour, 2024). Gas molecules undergo physical adsorption or chemical reactions with the sensing layer, altering the carrier concentration, mobility, or Schottky barrier height, resulting in a measurable change in resistance (Gao et al., 2019). This mechanism has a simple structure, is low-cost, and is easy to miniaturize. However, it is susceptible to humidity interference and suffers from baseline drift and long-term stability issues (Kim et al., 2019). In recent years, the selectivity and noise immunity of sensors have been significantly improved through surface functionalization, heterostructure fabrication, and composite materials engineering. For example, Li Z. et al. (2021) designed a wearable chemical resistance sensor based on AuNP@rGO composites for the real-time monitoring of the green leaf volatiles (E)-2-hexenal released from plant leaves, to assess mechanical damage and insect pest stress. The sensor exhibits a reversible response to (E)-2-hexenal at room temperature, with a detection limit of 5 ppm, and has been successfully applied to stress monitoring in tobacco plants. However, this study primarily focused on green leaf volatiles and did not cover terpenoid compounds, which are more prevalent in plant stress. Furthermore, the sensor’s sensitivity is relatively limited (producing only about a 3% change in resistance for a 10 ppm target gas), it has not yet been integrated with a wireless transmission module, and the impact of humidity on sensor performance has not been systematically evaluated. In contrast, building on the above work, Zheng et al., (2023b) designed a wearable chemical resistance gas sensor based on an Au interdigitated electrode and a polyethylene-co-vinyl acetate (PEVA)/MWCNTs composite sensing film for the real-time monitoring of the characteristic monoterpene α-pinene released by Chinese juniper plants under mechanical damage and drought stress. The sensor exhibits good selectivity toward α-pinene at room temperature, with a sensitivity of 8.32 kΩ/ppm (a resistance change of approximately 13% for 10 ppm of α-pinene) and a detection limit as low as 0.5 ppm. Furthermore, the design of the hydrophobic composite material renders the sensor insensitive to changes in relative humidity. In actual plant monitoring, the sensor resistance increased by approximately 15% following a 4mm bark injury and by approximately 2% after 40 days of drought stress. More importantly, this study integrated the sensor with a Near Field Communication (NFC) tag, enabling wireless, passive monitoring of plant stress. This addresses the reliance on wired measurements in previous studies by Li Z. et al. (2021) and further expands the application potential of chemical resistance sensors in precision agriculture and early diagnosis of plant health.

#### 3.2.3 Piezoelectric sensing mechanism

Piezoelectric sensing uses piezoelectric materials to convert the mechanical deformation caused by gas adsorption into a measurable electrical signal. This sensing mechanism relies on changes in the mass, viscoelasticity, or mechanical stress of piezoelectric materials when they interact with the target gas (Wen et al., 2015). Based on this mechanism, Wang et al. (2024a) reports a QCM sensor modified with MOF-74 material for CO_2_ detection at room temperature. This study leveraged the open metal sites of Mg-MOF-74 for the specific adsorption of CO_2_. By converting the mass loading into a decrease in resonant frequency via the Sauerbrey equation, the researchers achieved highly sensitive detection. This demonstrates the potential of piezoelectric sensing for the detection of inert gases at room temperature and provides a material foundation for the future development of flexible wearable devices. However, the sensor is extremely sensitive to minute mass changes, and improvements are still needed in terms of humidity sensitivity and long-term stability (Yang et al., 2025).

#### 3.2.4 Mechanical sensing mechanism

Mechanical sensors detect changes in resistance or capacitance by altering the electronic tunneling paths in a conductive network through gas-induced strain, stress, or deformation (Joshi et al., 2021). Based on this mechanism, Lee et al. (2023) reports a multimodal wearable sensor attached to the lower epidermis of plant leaves for continuous monitoring of plant physiological status. The sensor utilizes a composite conductive network composed of Au@AgNWs and MWCNTs as the VOCs sensing material. When VOCs molecules are specifically adsorbed by functional ligands on the surface of the nanowires, they induce microscopic strain in the conductive network and alter the electronic tunneling pathways, thereby causing a change in electrical resistance. The sensor successfully achieved real-time monitoring of VOCs released by tomato plants under conditions such as drought, salt stress, and pathogen infection, and detected diseases 2–3 days earlier than traditional detection methods, demonstrating the application potential of mechanical sensing mechanisms in wearable gas monitoring for plants. The sensor’s advantage lies in its high responsiveness to mechanical stimuli, however, its sensitivity, robustness, and resistance to environmental interference still require optimization (Wang et al., 2021).

#### 3.2.5 Optical sensing mechanism

Optical sensors monitor changes in optical parameters (such as absorption, fluorescence, refractive index, and reflectance) caused by the interaction between gases and the sensing layer (Afsari and Sarraf, 2020). This mechanism offers good selectivity and is free from electromagnetic interference, but its sensitivity is limited by the light source and detector; humidity interference and long-term optical stability also pose challenges (Wang et al., 2022). To address these challenges, researchers have developed various gas sensing platforms based on different optical mechanisms. For example, Nejad and Shekarchizadeh (2025) reported a dual-mode colorimetric/fluorescent indicator based on copper nanoparticles/nitrogen-doped carbon quantum dots (CuNPs/N@CQDs) on an agar hydrogel for the detection of ethylene gas during the ripening process of bananas. This sensor utilizes the interaction between ethylene and copper nanoparticles to induce a red shift in the localized surface plasmon resonance (LSPR) peak (from 369 nm to 446 nm), while simultaneously causing fluorescence quenching in the carbon quantum dots, thereby achieving a dual-mode colorimetric and fluorescent response. The indicator has a detection limit of 9.94 μM for ethylene, a sensitivity as high as 2.56 × 10^4^ nm/RIU, and exhibits good temperature stability and selectivity. This smart packaging indicator can be directly applied to the interior of food packaging, and the ripeness of fruit can be determined by observing color changes with the naked eye, without the need for external light sources or detectors, thereby avoiding the reliance on light sources and detectors inherent in traditional optical sensors. However, its response time is relatively long (approximately 120 minutes), and interference from water molecules may occur in high-humidity environments. In contrast, Yang et al. (2022) designed a smartphone-based optical fiber sensor (SOFS) for integration into wearable devices. It combines a first-generation Grubbs catalyst (PYG) fluorescent probe labeled with pyrene for the rapid detection of ethylene. The sensor utilizes the mechanism whereby the PYG probe releases a fluorophore following an alkene metathesis reaction with ethylene, resulting in fluorescence enhancement. Excited by a 365 nm laser, the fluorescence signal is transmitted via an optical fiber, and collected by a smartphone CMOS sensor. An in-house developed Android application analyzes the linear relationship between the B/G ratio in the RGB values and ethylene concentration (R^2^ = 0.9947 in the range of 0–350 ppm), enabling quantitative detection of ethylene. The system has a detection limit as low as 0.6 ppm and exhibits good selectivity toward common interfering gases (SO_2_, NO_2_, H_2_S, CO_2_, NH_3_) and VOCs. The SOFS system integrates a portable excitation light source, optical fiber transmission, and smartphone analysis. Its compact and lightweight design makes it easy to embed in wearable devices or use as a handheld terminal, making it suitable for rapid detection and real-time monitoring at fruit storage sites. However, the system still requires operation in a dark environment to minimize ambient light interference, and fiber alignment and optical stability need further optimization for long-term use.

#### 3.2.6 Sensing mechanism of field-effect transistors

Field-effect transistors (FET) sensors achieve highly sensitive detection by altering channelconductivity through the adsorption of gas molecules (Guo et al., 2023). Zhang et al. (2025) reported a flexible FET gas sensor based on a physically cross-linked chitosan gel. By introducing 1,3-propanediol to enhance the hydrogen-bond network and using graphene as the channel material, the sensor achieves highly sensitive detection of H_2_S at room temperature (sensitivity of 42.96 mV/ppm, detection limit of 0.5 ppm). The sensor utilizes the protonation reaction between the amino groups of chitosan and H_2_S, as well as the hydrogen-bond network to facilitate electron transfer. Combined with the ion-gel gate-controlled structure, it effectively suppresses baseline drift. Meanwhile, the flexible substrate and physically cross-linked network confer excellent mechanical stability and interference resistance, providing a viable solution for wearable gas sensing. The sensor’s advantages lie in its amplification effect and low power consumption. However, issues such as baseline drift, environmental interference, and stability must be addressed through surface modification and the use of composite materials (Yuvaraja et al., 2021). The selection of an appropriate sensing mechanism is highly dependent on the specific application requirements and environmental conditions. Metal oxide semiconductors (MOS) offer high sensitivity and fast response but typically require elevated operating temperatures (often >200°C), resulting in high power consumption and limited compatibility with flexible substrates (Pathak et al., 2023). Carbon-based nanomaterials, such as graphene, carbon nanotubes, and carbon black, provide excellent electrical conductivity, large specific surface areas, and room-temperature operability, yet they often suffer from limited selectivity and baseline drift under fluctuating humidity (Schroeder et al., 2019; Kim et al., 2019). Conductive polymers, including polyaniline and polypyrrole, are attractive for their tunable chemical functionality and mechanical flexibility, but generally exhibit lower sensitivity and long-term stability compared to inorganic counterparts (Armitage et al., 2020). Noble metal nanoparticles (such as Au, Pt, Pd, Ag) are frequently employed as catalytic sensitizers to enhance sensitivity and selectivity, though their high cost and potential agglomeration limit practical scalability (Rai et al., 2015). Metal-organic frameworks (MOFs) offer high porosity and tunable pore structures for selective gas adsorption, but their low electrical conductivity often requires hybridization with conductive materials for chemiresistive sensing applications (Yan et al., 2024; Wang et al., 2024b). To overcome the limitations of single-component materials, hybrid and composite strategies have been widely adopted. For example, combining carbon nanomaterials with metal nanoparticles or MOFs has demonstrated synergistic improvements in both sensitivity and selectivity (Li Z. et al. (2021); Zheng et al., 2023b; Lee et al., 2023). A critical trade-off exists between sensitivity and selectivity: highly sensitive materials often respond to a broad range of analytes, while highly selective materials may sacrifice sensitivity. Hybrid approaches, such as sensor arrays combined with pattern recognition algorithms, offer a promising strategy to address this trade-off by leveraging cross-responsive sensing elements and multivariate data analysis (Li Z. et al., 2021; Weerakoon et al., 2012). Therefore, the choice of sensing mechanism must balance the specific target analyte, detection environment, and practical constraints of field deployment.

### 3.3 Sensor fabrication and integration technology

The fabrication of the sensor refers to the preparation and integration of its three-layer sandwich structure. As a core technical bottleneck that determines the performance consistency, long-term operational stability, and feasibility of large-scale industrial production of wearable gas sensor devices, the selection of fabrication techniques and process optimization directly impact the implementation and application of these sensors in scenarios involving *in-situ* monitoring of plant health. In the research and development process of wearable gas sensors for *in-situ* plant monitoring, existing fabrication technologies focus on four core objectives of “flexible adaptability, sensing sensitivity, scalability, and environmental tolerance”, which can be categorized into five major technical systems. Based on different mechanisms of action and material compatibility characteristics, each system demonstrates unique technical advantages and application scopes in sensor construction.

#### 3.3.1 Solution processing technology

Solution processing technology, as the most fundamental and widely used strategy for preparing sensor active layers, encompasses various specific methods including drop-casting (Xu et al., 2018; Gao et al., 2020), dip-coating (Owyeung et al., 2019), spin-coating (Araki et al., 2017; Li et al., 2017), blade-coating (Lin et al., 2019), spray-coating (Ho et al., 2016), and immersion coating (Kim et al., 2017). The core principle of this technology lies in utilizing the solubility of materials in solvents to achieve the integration of active sensing layers with flexible substrates through processes such as physical spreading, wetting, or centrifugal film formation. It offers notable advantages, including simple operational procedures, low equipment costs, and strong process compatibility, making it particularly suitable for film formation and device fabrication using soluble nanomaterials (such as carbon nanotubes, graphene, and conductive polymers). In the fabrication of plant-based wearable sensors, solution processing technology, with its mild reaction conditions, effectively prevents structural damage to the flexible substrates required for *in-situ* plant monitoring (such as PDMS, PU, and flexible paper substrates). Additionally, by adjusting solution concentration, coating speed, and substrate wettability, it enables precise control over the thickness and uniformity of the sensing layer, providing an efficient solution for the low-cost, rapid prototyping of sensor devices. However, this technology still faces certain limitations regarding film density, swelling stability during long-term use, and consistency control in mass production. A representative plant-wearable gas sensor fabricated via solution processing was reported by Li Z. et al. (2021). They employed a combination of stencil dip-coating and spin-coating to deposit reduced graphene oxide (rGO) and functionalized gold nanoparticle composites onto polyimide (PI) substrates, forming an 8-channel chemiresistive sensor array for real-time monitoring of 13 types of plant VOCs. In terms of application suitability, solution processing offers excellent compatibility with various flexible substrates and enables precise thickness control, which is beneficial for achieving conformal contact with leaf surfaces. However, the resulting films may exhibit limited gas permeability and humidity stability due to the dense polymer matrix, and the scalability for batch production remains constrained by inconsistent film uniformity across large areas.

#### 3.3.2 Vacuum deposition technology

Vacuum deposition technology is a key process for fabricating high-purity, high-performance flexible sensor active layers, and primarily comprises two major branches: physical vapor deposition (PVD) and chemical vapor deposition (CVD) (Li et al., 2017). PVD technology includes DC sputtering (Raghu et al., 2019), total evaporation/thermal evaporation (Chen et al., 2018), electron beam evaporation (Lee et al., 2016) and other specific embodiments. The core mechanism of this technology lies in the atomic-level deposition of materials onto flexible substrates through atomic/molecular evaporation, sputtering, or chemical reactions in a vacuum environment, enabling the fabrication of high-purity, highly uniform, and highly dense single-crystal or polycrystalline thin films. In the fabrication of plant-based wearable gas sensors, vacuum deposition technology can be used to construct high-performance metal electrodes and metal oxide sensing layers (such as ZnO, SnO_2_, WO_3_, etc.), significantly enhancing the sensor’s adsorption capacity and response sensitivity toward target gases such as plant VOCs and ethylene. Furthermore, by integrating with flexible substrate transfer technology, high-performance sensing films can be efficiently laminated onto flexible substrates, resulting in wearable sensor devices with excellent bending performance and environmental tolerance. However, vacuum deposition technology faces challenges such as high equipment costs, stringent process conditions (requiring a high-vacuum environment), and significant interfacial stress between the substrate and the film. These issues have limited their direct application in the low-cost, large-scale fabrication of plant sensors to some extent. Despite its high cost, vacuum deposition has been effectively applied in plant-wearable gas sensors. Lee et al. (2023) employed spray-coating combined with transfer printing (technically bridging vacuum-deposited metal films with flexible substrates) to fabricate Au@Ag core-shell nanowire and MWCNT-based multimodal sensors on PDMS substrates. The vacuum-deposited metal layers ensured high electrical conductivity and uniform electrode patterns, which are critical for reliable VOC detection. From a deployment perspective, vacuum-deposited sensors exhibit strong mechanical durability and stable baseline resistance under bending cycles, but the rigid metal films may slightly compromise conformal adhesion to growing leaf surfaces. Furthermore, the high equipment cost and batch-to-batch variability limit their scalability for cost-sensitive agricultural applications.

#### 3.3.3 Printing and manufacturing technology

Printing and manufacturing technologies are used to integrate sensing active layers with flexible substrates. As a core technological approach for the mass production of flexible electronics, it mainly includes mainstream methods such as inkjet printing (Hondred et al., 2018), screen printing (Araki et al., 2017) and extrusion printing (Wang et al., 2024b). Its core value lies in leveraging precise patterning control methods to achieve high-precision, large-area, and low-cost fabrication of conductive electrodes, sensitive material layers, and flexible substrates, thereby overcoming the limitations of traditional fabrication technologies in terms of patterning accuracy and production efficiency. Inkjet printing technology achieves precise material deposition through microdroplet ejection. Characterized by template-free, non-contact processing, it allows for precise control over the distribution of sensing materials and pattern dimensions, making it suitable for constructing miniaturized, integrated plant-based wearable sensor arrays. Screen printing technology, leveraging its stencil-based advantages, enables the rapid, large-area transfer of high-viscosity conductive inks and sensing materials. It offers high production efficiency and controllable costs, making it suitable for the mass production of electrodes and sensing layers on flexible substrates. Extrusion printing technology is compatible with the processing of various viscous materials, enabling the uniform fabrication of thick-film sensing layers, thereby enhancing the sensor’s sensitivity and stability in responding to plant gas signals. In the fabrication of plant-wearable sensors, these technologies enable the integrated fabrication of sensing units with flexible substrates and are compatible with various flexible substrate materials, such as textiles and films. They provide the technical foundation for constructing large-area, arrayed *in-situ* plant health monitoring networks. However, further optimization is still needed in terms of the uniform deposition of nanomaterials, printing resolution, and the control of substrate adhesion. Printing technologies have shown particular promise for plant-wearable gas sensors. Wang et al., (2024b) utilized extrusion printing to fabricate dye/UiO-66(Zr)@PDMS composite sensors on flexible PDMS films for colorimetric VOC detection, achieving a detection limit of 0.5–1 ppm with a response time of approximately 20 minutes under high-humidity conditions (90% RH). The extrusion-printed sensors maintained functional integrity even under mechanical bending, demonstrating robust interfacial adhesion. Notably, printing technologies offer distinct advantages for agricultural deployment: they enable low-cost, high-throughput production with consistent pattern reproducibility; the porous nature of printed sensing layers facilitates rapid gas diffusion and high sensitivity; and the compatibility with roll-to-roll processes makes large-scale manufacturing feasible. However, the long-term humidity stability and resistance to UV degradation of printed organic-inorganic composite inks remain challenging for extended field deployment.

#### 3.3.4 *In-situ* growth/polymerization technology

*In-situ* growth/polymerization technology is a key technical approach for achieving a strong bond between the active sensing layer and a flexible substrate, typically exemplified by hydrothermal growth (Yang et al., 2019) and *in-situ* polymerization (Wang et al., 2018; Shimpi et al., 2015). The core advantage of this technology lies in its elimination of traditional post-processing lamination processes. Instead, it enables the *in-situ* growth and polymerization of functional active materials directly on the surface of a flexible substrate through chemical reactions. This effectively enhances the interfacial bonding strength between the sensing materials and the substrate, thereby improving the sensor’s long-term stability and peel resistance during *in-situ* plant monitoring. In the fabrication of plant-wearable sensors, hydrothermal growth technology can be used to *in situ* grow sensitive materials, such as nanoscale metal oxides and carbon materials, on the surface of flexible substrates by controlling reaction temperature, time, and precursor concentration. This creates porous sensing structures with large specific surface areas, thereby enhancing the sensor’s adsorption and response characteristics toward target gases. *In-situ* polymerization technology, on the other hand, allows for the direct polymerization of conductive polymers (such as PPy and PANI) on the surface of flexible substrates, producing sensing layers with excellent flexibility and conductivity that meet the design requirements for stretchable and bendable plant-worn sensors. Although this technology involves a relatively complex operational process and requires high precision in controlling reaction conditions, its unique advantages in enhancing sensor interface stability and sensing performance make it a key technological choice for the fabrication of *in-situ* plant monitoring sensors designed for complex field environments. *In-situ* growth and polymerization strategies have been leveraged to enhance sensor adhesion and stability on plant surfaces. Kim et al. (2026) developed an amphibian toe pad-mimicking wearable gas sensor by combining PDMS molding with electrochemical deposition and transfer molding, physically embedding MWCNT electrodes into the PDMS substrate. The *in-situ* growth approach ensured strong interfacial bonding between the sensing material and the substrate, preventing delamination even after 10 washing cycles. This technique excels in mechanical durability and resistance to detachment caused by leaf growth or surface expansion. However, the relatively complex fabrication process and stringent reaction conditions limit its scalability for mass production. Additionally, the dense interfacial bonding may reduce gas permeability to some extent, requiring careful design of porous sensing structures.

#### 3.3.5 Emerging micro/nano manufacturing processes

Emerging micro/nano manufacturing processes offer innovative technical solutions for the flexible, multifunctional, and structured design of plant-based wearable sensors, mainly including innovative technologies such as layer-by-layer self-assembly (Su and Liao, 2019), femtosecond laser direct writing (An et al., 2017), electrospinning (Cerón et al., 2016), traditional embroidery (Seesaard et al., 2015) and wet spinning (Zhou et al., 2017). Layer-by-layer self-assembly technology relies on electrostatic interactions or chemical bonding between molecules. By alternately depositing various functional materials, it enables the precise construction of multi-level, multifunctional sensing structures. This approach allows for the fabrication of wearable sensors with multi-layer sensing capabilities, enabling the simultaneous monitoring of various gas signals from plants. Femtosecond laser direct writing technology, leveraging the high-precision processing capabilities of ultrashort-pulse lasers, enables the precise etching and material modification of micro- and nanostructures on flexible substrates. This facilitates the construction of miniaturized, highly sensitive sensing units, meeting the development requirements for miniaturized *in-situ* plant monitoring sensors. A representative example of electrospinning for plant-wearable sensing platforms was recently demonstrated by Zhu et al. (2024). They fabricated an ultrathin self-healing nanofibrous membrane with a hierarchical confined structure as a biomimetic epidermal electrode substrate. The electrospun membrane exhibited exceptional breathability, mechanical compliance, and room-temperature self-healing capability. Notably, this nanofibrous membrane was successfully attached to pea seedling surfaces for *in situ*, long-term monitoring of plant growth signals, validating its practical applicability in plant-wearable systems. The ultrathin thickness and porous network structure minimized mechanical interference with plant tissues and ensured unimpeded gas exchange through stomata, while the self-healing property effectively prolonged device lifespan under dynamic leaf growth conditions. This work highlights the significant potential of electrospinning for constructing high-performance, plant-compatible flexible sensor substrates. Electrospinning technology can produce sensor materials with one-dimensional nanofiber structures, characterized by large specific surface area, high porosity, and excellent flexibility. This significantly enhances the sensor’s gas adsorption and response efficiency while facilitating the fabrication of flexible sensors on textile substrates. Traditional embroidery techniques and wet spinning technologies fully leverage the characteristics of textile substrates commonly used in agricultural settings. By integrating sensing materials into textile substrates via embroidery processes or by fabricating sensing fibers through wet spinning, these methods enable the development of textile-based plant wearable sensors with excellent wearability and environmental adaptability. These sensors are particularly suitable for large-scale, long-term field plant monitoring applications. For agricultural deployment, electrospinning offers excellent gas permeability due to its porous architecture, which facilitates rapid VOC diffusion to active sensing sites. The nanoscale features, however, may exhibit limited mechanical robustness under repeated leaf motion or rain exposure, and the long-term stability of electrospun membranes under fluctuating temperature and humidity requires further validation. Nevertheless, the scalability of electrospinning shows promise for cost-effective deployment over large agricultural areas.

In summary, the fabrication processes for wearable gas sensors designed to meet the demand for continuous *in-situ* monitoring of plant health are showing a trend toward “diversification, differentiation, and synergy”. Based on their respective mechanisms and characteristics, different technological systems have their own strengths in terms of sensor performance tuning, substrate adaptation, cost control, and mass production. Through the synergistic optimization and integrated innovation of multiple processes, such as the combination of solution processing and printing technologies, the complementarity of *in-situ* growth and vacuum deposition technologies, and the convergence of emerging micro/nano fabrication processes with traditional fabrication techniques, it is possible to effectively overcome the key technological bottlenecks in transitioning sensors from laboratory prototypes to large-scale flexible integration, low-cost mass production, and stable field applications. This provides core technological support for promoting the industrial application of wearable gas sensors in the field of *in-situ* plant health monitoring and the development of smart agriculture.

## 4 Applications of wearable gas sensors

### 4.1 Detection of environmental stress in growth conditions

#### 4.1.1 Drought stress

**Figure 4:**
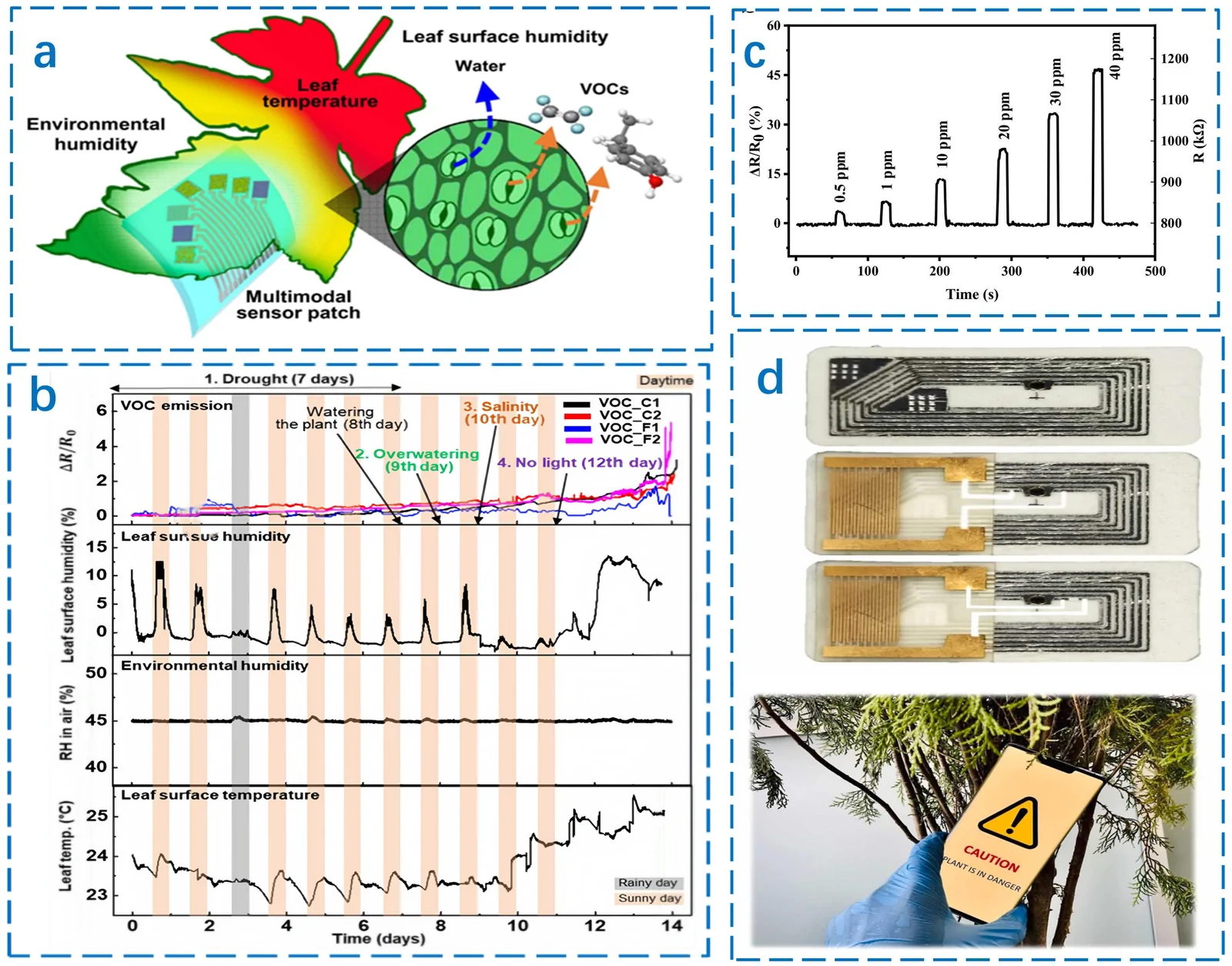
**(a)** Schematic diagram of sensors mounted on plant leaves (Lee et al., 2023); **(b)** Real-time sensor data from the same tomato plant exposed to various abiotic stresses (Lee et al., 2023); **(c)** Response-recovery curve of the sensor at α-pinene concentrations ranging from 0.5 to 40 ppm (Zheng et al., 2023b); **(d)** Commercial NFC tags and their parallel and series integration with the IDE sensor, as well as the detection of mechanical damage using an NFC-enabled smartphone (Zheng et al., 2023b).

Drought is one of the primary yield-limiting factors in global agriculture. Water deficit triggers a series of cascading reactions, including stomatal closure, reduced transpiration, inhibited photosynthesis, massive accumulation of ABA, and bursts of reactive oxygen species (ROS), ultimately affecting cell elongation, root development, and the formation of reproductive organs (Oyedoh et al., 2025; Oguz et al., 2022; Rai et al., 2024). Lee et al. (2023) fabricated a PDMS-based multimodal wearable sensor mounted on leaf abaxial stomatal-rich surfaces, integrating VOC chemiresistors and humidity/temperature detectors (**Figure 4a**). A 3D nano-hybrid network suppresses signal crosstalk, with machine learning for data processing. Attaching beneath leaves boosts VOC and thermal/humidity signals by ~40%, allowing quantitative discrimination of drought, salt and pathogen stress; the device alerts tomato drought 3–5 d earlier and sustains 14-day stable testing, overcoming defects of single-parameter detection (**Figure 4b**). Nevertheless, bulky dimensions induce mechanical damage to tender foliage, absent wireless circuitry confines field large-scale use, and verification is restricted only to tomato rather than field crops. Regarding the targeted detection of VOCs and the wireless and practical implementation of wearable sensors, Zheng et al., (2023b) fabricated an Au-IDE wearable chemiresistor with PEVA/MWCNT sensitive layer for selective α-pinene (drought biomarker of Chinese juniper), reaching an LOD of 0.5 ppm and sensitivity of 8.32 kΩ/ppm (**Figure 4c**). The hydrophobic sensor works steadily at room temperature. Combined with NFC tags, the passive wireless device realizes battery-free field monitoring, identifying 2% α-pinene variation under 40-day drought and 15% signal shift upon mechanical injury (**Figure 4d**). Differing from Lee’s multi-parameter sensor, this single-VOC detector features low cost and facile fabrication. Still restricted by single-gas detection, it fails to differentiate drought from mechanical damage, shows faint responses to mild drought, and is only available for α-pinene-releasing conifers. The drought stress monitoring examples discussed above highlight a notable gap between laboratory-based sensor characterization and field-deployable performance. Lee et al. (2023) demonstrated 14-day continuous monitoring under controlled growth chamber conditions, yet the sensor’s bulky dimensions and absence of wireless data transmission render it impractical for large-scale field deployment. Zheng et al., (2023b) addressed the wireless transmission challenge through NFC integration, but the single-VOC detection strategy suffers from limited diagnostic specificity under real-world conditions where multiple stresses coexist. While laboratory studies typically report impressive sensitivity (LOD as low as 0.5 ppm for α-pinene) and rapid response times, field conditions introduce confounding factors, including fluctuating temperature and humidity, background VOC interference, and sensor fouling, that substantially degrade performance. Moreover, the mechanical integrity of flexible sensors under repeated leaf motion, rain exposure, and UV radiation remains underexplored, with most studies reporting bending stability tests only under benign laboratory conditions rather than realistic agricultural environments. These disparities underscore the critical need for standardized field validation protocols and long-term stability assessments to bridge the gap between proof-of-concept demonstrations and practical agricultural applications.

#### 4.1.2 Salt stress

**Figure 5:**
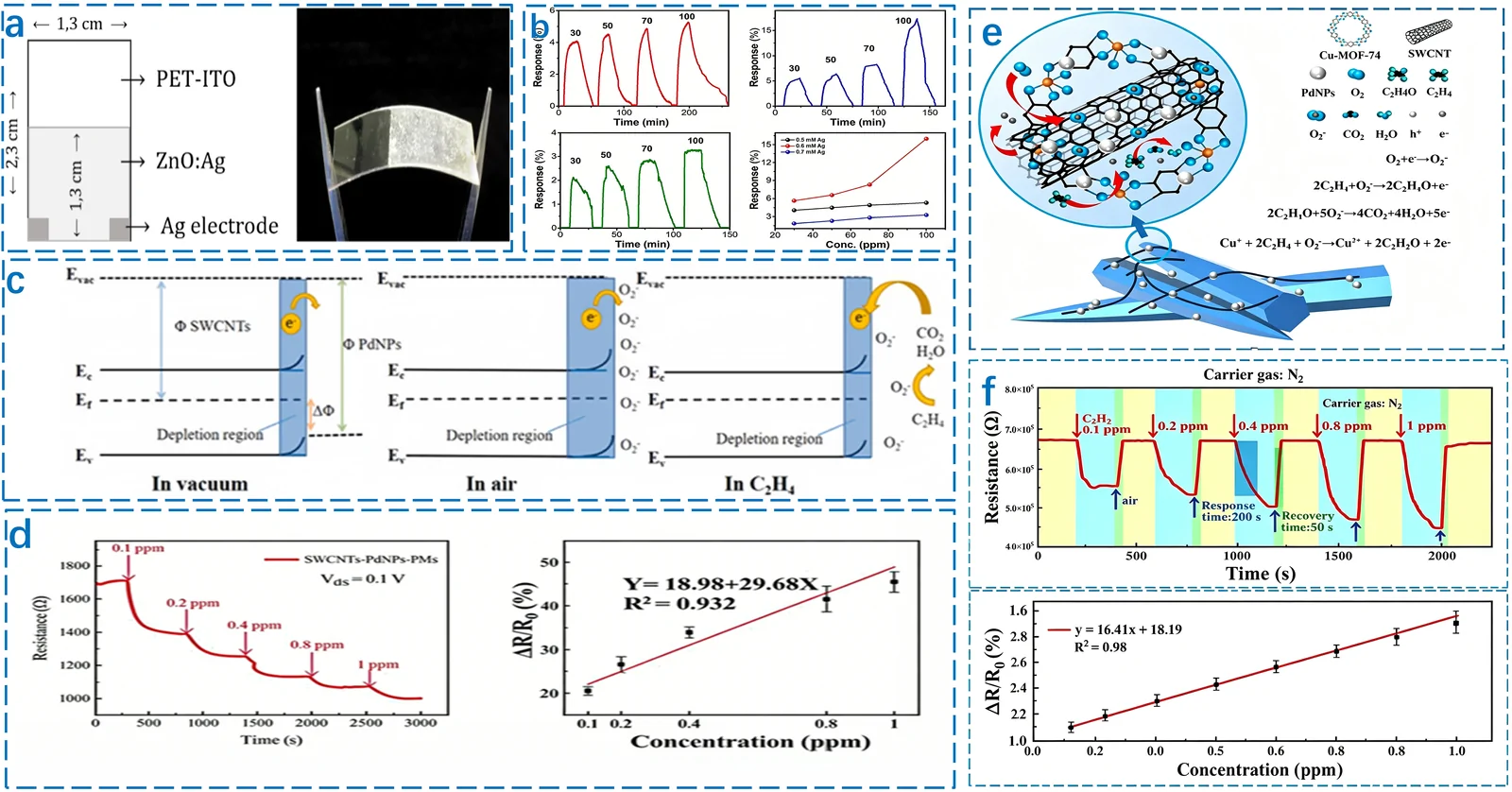
**(a)** ZnO-Ag ethylene sensor: schematic diagram of the principle (left) and sensor prototype (right) (Sholehah et al., 2021); **(b)** Sensor response curves: ZnO-Ag 0.5 (top left), ZnO-Ag 0.6 (top right), and ZnO-Ag 0.7 (bottom left); sensitivity curves for all samples (bottom right) (Sholehah et al., 2021); **(c)** Schematic diagram of the C_2_H_4_ sensing mechanism, explained using the Schottky barrier under (left) vacuum, (middle) air, and (right) C_2_H_4_ conditions (Yan et al., 2023); **(d)** Stepwise detection curves for ethylene in the concentration range of 0.1 ppm–1 ppm (left) and the corresponding linear fitting equations for ethylene in the same range (right) (Yan et al., 2023); **(e)** Schematic illustration of the sensing mechanism of the SPM/AIEFP sensor (Yan et al., 2024); **(f)** Real-time resistance variation curves of ethylene (left) and the corresponding linear fitting equations (right) at concentrations ranging from 0.1 ppm to 1 ppm (Yan et al., 2024).

Salt stress severely inhibits plant growth through ion toxicity, osmotic stress, and oxidative damage. As a key stress hormone, ethylene plays a dual role in salt adaptation: low concentrations promote salt tolerance, whereas high concentrations induce senescence (Tao et al., 2024). Sholehah et al. (2021) electrodeposited a ZnO-Ag composite layer on a PET-ITO flexible substrate (**Figure 5a**), achieving effective detection of 29 ppm ethylene at room temperature with a response range of 17.2%–19.6% and a recovery time of only 4–8 min (**Figure 5b**). Yan et al. (2023) developed a paper-based sensor composed of SWCNTs-PdNPs-polystyrene microspheres, which lowered the detection limit of ethylene to 100 ppb (**Figures 5c, d**). The further optimized PdNPs-SWCNTs@Cu-MOF-74 composite improved the detection limit to 31 ppb and reduced the response and recovery times to 200 s and 50 s, respectively (**Figures 5e, f**). It has a service life of up to 13 days and remains stable within a humidity range of 40%-70% (Yan et al., 2024). These wearable sensors optimize sensitivity, selectivity, and flexibility adaptability through material composite strategies, enabling *in-situ* capture of ethylene emission signals during the early stage of plant salt stress. They provide real-time data support for crop salt damage early warning and precision irrigation, significantly expanding the application scope of wearable sensing technology in agricultural stress monitoring (Jiang et al., 2013; Deinlein et al., 2014). Furthermore, although the Cu-MOF-74 structure enhances selectivity for ethylene, prolonged exposure to air may cause the MOF structure to collapse, thereby reducing the service life of the sensor. Therefore, future research should focus on developing humidity-adaptive encapsulation layers to improve the structural stability of MOF materials, and integrate multi-channel sensor arrays to distinguish ethylene from other interfering gases (such as CO_2_ and ethanol), thereby enhancing the reliability and practicality of the sensor in complex agricultural environments. The salt stress detection studies also illustrate the performance gap between laboratory-controlled conditions and agricultural environments. Yan et al. (2023) achieved an impressive LOD of 100 ppb for ethylene detection on paper-based substrates under laboratory conditions, yet the sensor’s response degraded significantly when relative humidity exceeded 70%, a common condition in many agricultural settings. Similarly, the ZnO-Ag sensor developed by Sholehah et al. (2021) demonstrated room-temperature ethylene detection on flexible PET-ITO substrates, but its recovery time of 10–15 minutes and LOD of 30 ppm, while acceptable for laboratory characterization, are insufficient for early salt stress warning in field applications where rapid and sensitive detection is critical. Furthermore, the long-term stability of these sensors under continuous field deployment remains a concern: Yan et al. (2024) reported a service life of only 13 days for PdNPs-SWCNTs@Cu-MOF-74 sensors before performance degradation, which is inadequate for a full growing season. These examples highlight that while material innovations have significantly improved laboratory performance, translating these advancements into robust, long-lasting field sensors requires further attention to environmental durability, operational lifespan, and real-world validation.

#### 4.1.3 Nutritional deficiency screening

**Figure 6:**
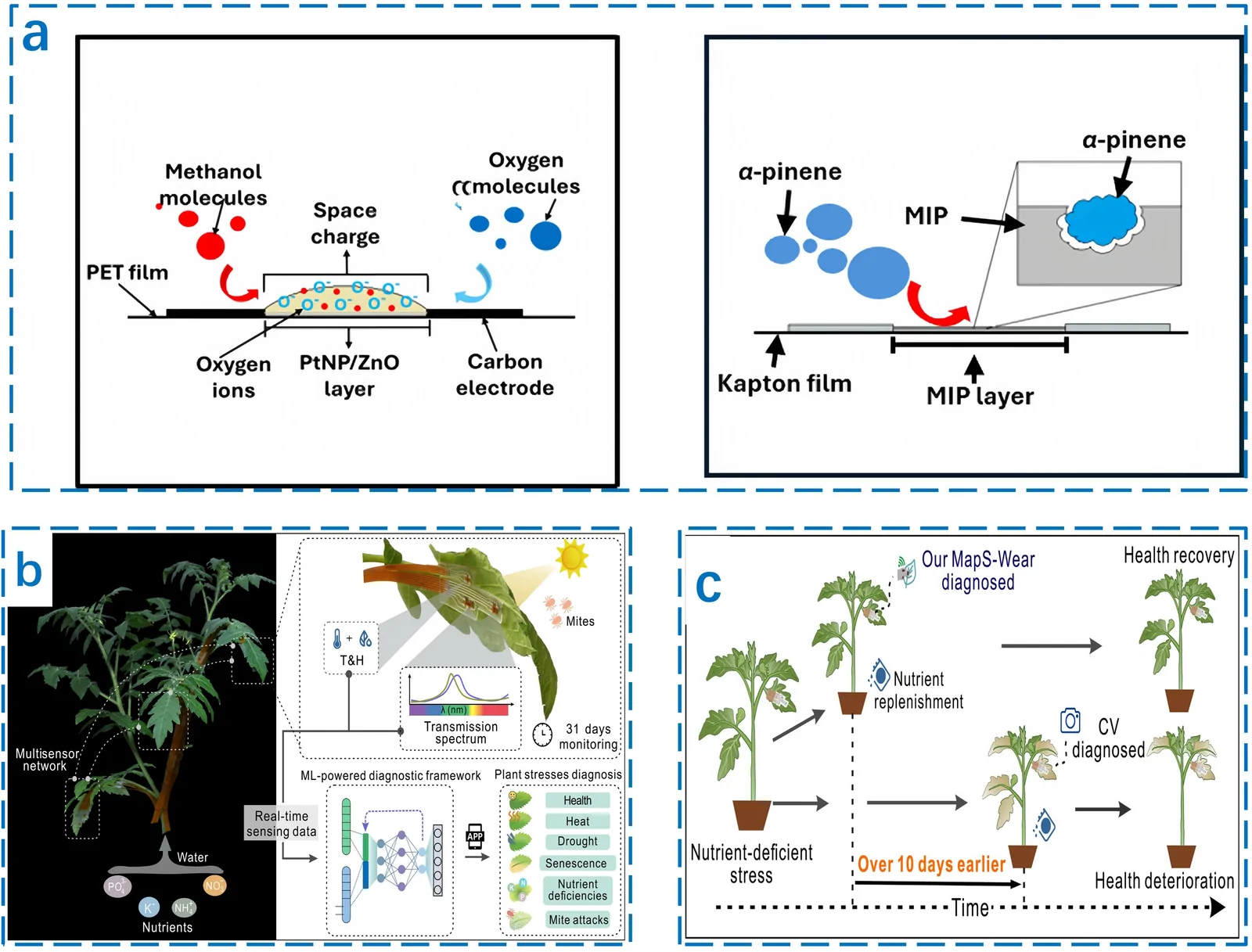
**(a)** *In-situ* dual-gas sensing system: schematic diagram illustrating the principles of the methanol (left) and α-pinene (right) sensors (Sangmen et al., 2026); **(b)** MapS-Wear for long-term and *in-situ* diagnosis of plant stress (Jiang et al., 2025); **(c)** MapS-Wear’s ability to diagnose nutrient deficiency symptoms at an early stage (Jiang et al., 2025).

Nutrient stress in plants (nitrogen, phosphorus, or potassium deficiency) triggers the release of characteristic VOCs through metabolic disturbances. Leveraging their core advantages of *in situ*, non-invasive, and long-term monitoring, wearable sensors have emerged as a cutting-edge technology for the precise diagnosis of nutrient stress. Their development is grounded in the fundamental mechanisms of plant stress responses and benefits from the innovative integration of sensing technologies and data analysis methods. As the second-largest oilseed crop worldwide, the yield and quality of rapeseed and other major crops are not only threatened by abiotic stresses such as high temperatures and drought but are also frequently compromised by nutrient imbalances. As the primary organ for sensing plant stress, the root system transmits its response to nutrient deficiency through metabolic pathways to the aboveground parts, inducing the release of characteristic VOCs such as terpenes, aldehydes, and nitrogen- and sulfur-containing compounds (Wu et al., 2018). Breakthroughs in modern nanobiosensing technology provide critical support for the precise detection of this signal. Singh et al. (2026) systematically reviewed the innovative applications of advanced wearable platforms such as tattoo-like sensors, smart packaging sensors, and microneedle arrays. Flexible sensing interfaces constructed using nanomaterials like MXene and carbon nanotubes can achieve ppm-level detection of nutrient stress-related VOCs such as methanol and α-pinene, with response times of less than 20 s. Furthermore, biocompatible designs ensure long-term monitoring without physiological interference, perfectly meeting the needs of dynamic plant nutrient tracking. For example, Sangmen et al. (2026) recently developed a dual-sensor platform by integrating metal oxide nanoparticles and molecularly imprinted polymers onto a flexible substrate, which achieved *in-situ*, real-time and continuous detection of methanol and α-pinene released from cotton leaves under nitrogen deficiency stress (**Figure 6a**), providing crucial evidence for the early diagnosis of nutrient stress. However, the wearable sensing technology has some limitations, such as fewer signals that can be collected, lack of specificity, and high susceptibility to environmental interference. Jiang et al. (2025) developed a machine learning-driven spectral-VOC multimodal flexible wearable system (MapS-Wear) (**Figure 6b**). By integrating flexible gas sensing with hyperspectral imaging, it achieved accurate capture of nutrient stress-induced VOCs and cross-validation of multimodal features. This not only improved the diagnostic accuracy for nitrogen, phosphorus, and potassium deficiencies to 99.2%, but also enabled stress early warning 10 days in advance (**Figure 6c**). This overcomes the limitation that a single VOC signal is easily confused with other stressors. Notably, Sangmen et al. (2026) further integrated VOCs sensing with physiological parameters such as chlorophyll indices and stomatal conductance, as well as continuous monitoring of soil nitrate levels. It revealed significant correlations between the emission intensities of methanol and α-pinene and chlorophyll degradation and stomatal regulation, thereby laying the physiological foundation for the development of a multi-parameter integrated model for diagnosing nutrient stress. Chen et al. (2023) further note in their review that the core areas of development for wearable plant sensors lie in material innovation (such as MXene and conductive polymers), multi-signal fusion, and the optimization of AI algorithms, Furthermore, the study by Singh et al. (2026) has further refined the practical approach to multi-parameter integration. By combining VOCs detection with nutrient ion (NO_3_^-^, K^+^) sensing and coupling with machine learning algorithms, accurate classification of stress types was achieved with an accuracy exceeding 97%, thereby confirming the core value of VOCs as “molecular messengers” reflecting plant nutrient metabolic status. Such technological integration has emerged as a major breakthrough for precision agriculture. In the future, it will be necessary to further integrate the physiological mechanisms of plant stress responses (such as root-to-shoot signal transduction pathways), draw on the principles of green nanotechnology to optimize the environmental durability and biocompatibility of sensors, and simultaneously enhance the detection sensitivity for low-concentration characteristic VOCs. This will drive the transition of nutrient stress diagnosis from the laboratory to large-scale field applications, providing technical support for closed-loop fertilization and high-yield, high-quality crop production. Notably, the nutritional deficiency monitoring studies illustrate both the promise and the remaining challenges of translating wearable sensors to precision agriculture. Sangmen et al. (2026) conducted one of the few field-validated studies, demonstrating 23-day continuous monitoring of methanol and α-pinene emissions from cotton plants under greenhouse conditions. Their dual-sensor platform successfully captured the elevated VOC emissions (17.65 ppm methanol, 1217.49 ppb α-pinene) associated with nitrogen deficiency, with statistical significance (p < 0.0001). However, even this field-validated study revealed important limitations: humidity compensation was performed through post-processing rather than real-time on-board correction, and the sensor ‘s response exhibited moderate correlation coefficients (R² = 0.2789 for methanol vs. chlorophyll index), indicating that VOC emissions are influenced by multiple physiological and environmental factors beyond nutrient status alone. Jiang et al. (2025) achieved impressive 99.2% diagnostic accuracy for nutrient deficiency using a multimodal system combining VOC sensing with hyperspectral imaging, yet the system complexity and cost currently limit its scalability for routine agricultural use. These studies collectively suggest that while wearable VOC sensors hold significant potential for nutrient stress diagnosis, their practical deployment requires further simplification, cost reduction, and integration with environmental compensation mechanisms to account for confounding variables inherent in field conditions.

### 4.2 Detection of pest and disease stress

#### 4.2.1 Disease detection

**Figure 7:**
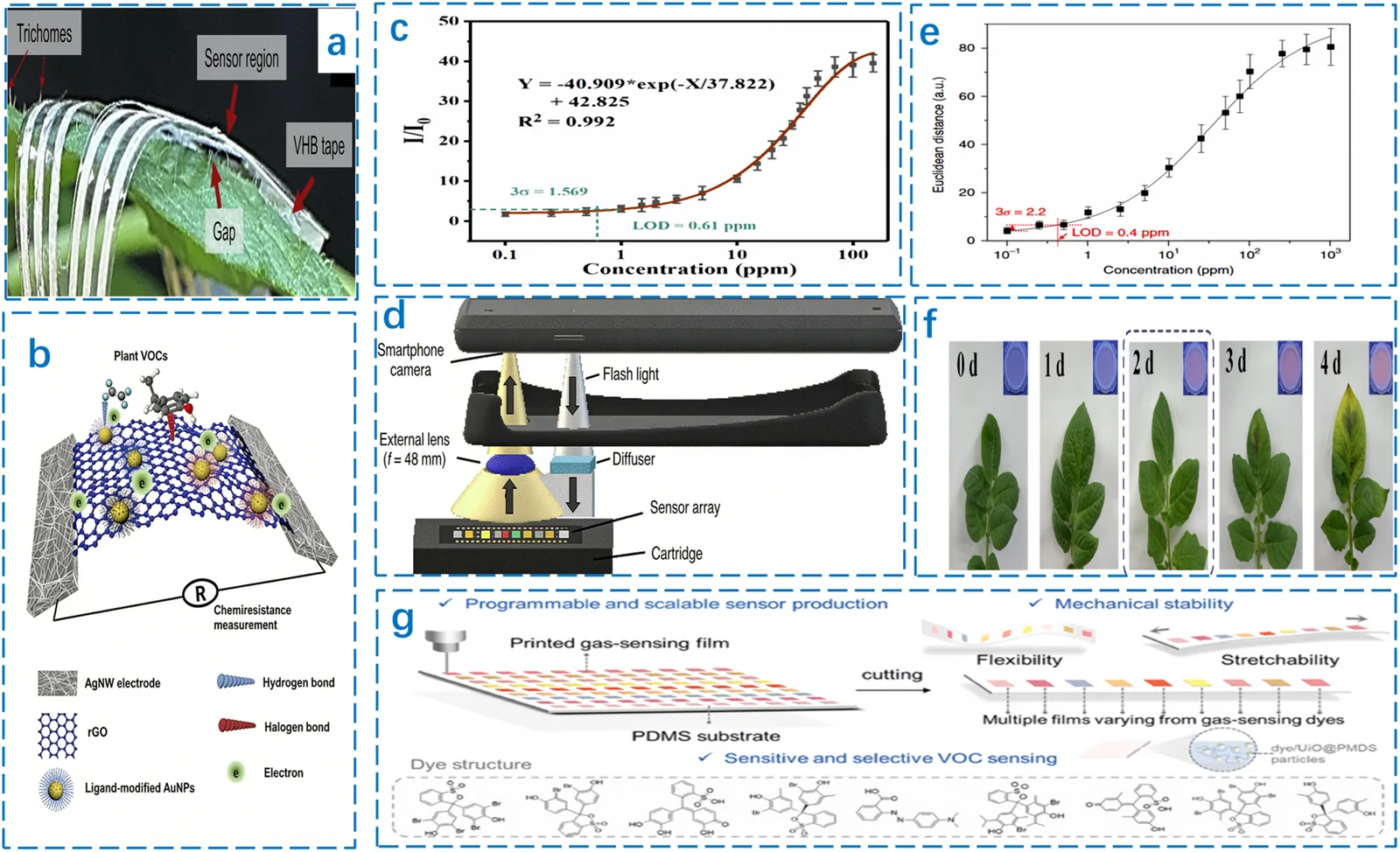
**(a)** Photograph of the tomato sensor installation (Li Z.et al., 2021); **(b)** Schematic diagram of a wearable sensor utilizinggraphene-based sensing materials and flexible AgNW electrodes (Li Z.et al., 2021); **(c)** Graph illustrating the relationship between I/I0 and(E)-2-hexenal concentration (Li Z. et al., 2019); **(d)** Schematic diagram of a sensor array scanning a smartphone device (Gan and Wang, 2024); **(e)** Response curve of the Cys-Au NR sensor to (E)-2-hexenal (Li Z. et al., 2019); **(f)** Monitoring of (E)-2-hexenal released from potato leaves following inoculation with late blight pathogen (P. infestans) using fluorescent hydrogel imaging technology (Wang et al., 2024b); **(g)** Flexible, stretchable sensor arrays for the visual detection and identification of VOCs (Wang et al., 2024b).

Characteristic VOCs (such as (E)-2-hexenal) released in the early stages of plant diseases are key to non-destructive diagnosis. Li Z. et al. (2021) developed an rGO-based chemical resistance sensor array (**Figures 7a, b**). Functional modification enabled highly accurate classification (>97%) of 13plant-derived VOCs and allowed for early detection 4 days after inoculation with tomato late blight. A subsequently developed smartphone-integrated colorimetric array (Li Z. et al., 2019) achieved a detection limit of 0.4 ppm for (E)-2-hexenal, with a field blind test accuracy of 97.5%, and was capable of distinguishing between late blight and early blight (**Figures 7d, e**). Gan and Wang (2024) proposed a fluorescent hydrogel assay kit based on AIE-Au NCs, which achieved quantitative detection at 0.61 ppm via the Michael addition reaction and enabled identification prior to the appearance of late blight symptoms in potato (**Figures 7c, f**). The multimodal sensor developed by Lee et al. (2023) (**Figure 4b**) integrates multiple parameters, including VOCs, temperature, and humidity, and successfully distinguishes between viral infection and abiotic stress using machine learning algorithms. Wang et al., (2024b) prepared colorimetric arrays using extrusion-printed MOF/PDMS composite ink (**Figure 7g**). It maintains a detection limit of 0.5–1 ppm even in high-humidity environments and enables early diagnosis two days after inoculation with wheat fusarium head blight. These advancements mark a shift in disease monitoring from single-parameter to multimodal, intelligent approaches. Despite these impressive advances, the translation of disease-detecting wearable sensors from laboratory validation to field deployment faces several challenges. The chemiresistive sensor array developed by Li Z. et al. (2021) demonstrated early detection of tomato late blight 4 days post-inoculation under controlled growth chamber conditions, with >97% classification accuracy for 13 plant VOCs. However, field conditions introduce additional complexities: diurnal temperature and humidity variations, natural VOC background from neighboring plants, and sensor biofouling from airborne particulates and leaf surface exudates all contribute to signal degradation that is rarely encountered in laboratory settings. Similarly, Gan and Wang (2024) achieved remarkable 0.61 ppm LOD for (E)-2-hexenal using a fluorescence-based hydrogel kit, with detection 2 days post-inoculation on detached potato leaves. While this portable kit offers simplicity and low cost, its irreversible colorimetric response and reliance on detached leaf samples limit its applicability for continuous, non-destructive monitoring of living plants. Wang et al., (2024b) demonstrated *in situ* wheat scab detection 2 days post-inoculation using extrusion-printed colorimetric sensor arrays, representing one of the few truly field-validated disease detection studies. Nevertheless, the 20-minute response time and the requirement for scanner-based image capture, rather than integrated wireless readout, remain practical constraints for widespread field adoption. These examples underscore that while laboratory studies have demonstrated the feasibility of VOC-based disease detection, achieving robust field performance requires addressing environmental interference, sensor regeneration, and user-friendly data acquisition.

#### 4.2.2 Pest stress

**Figure 8:**
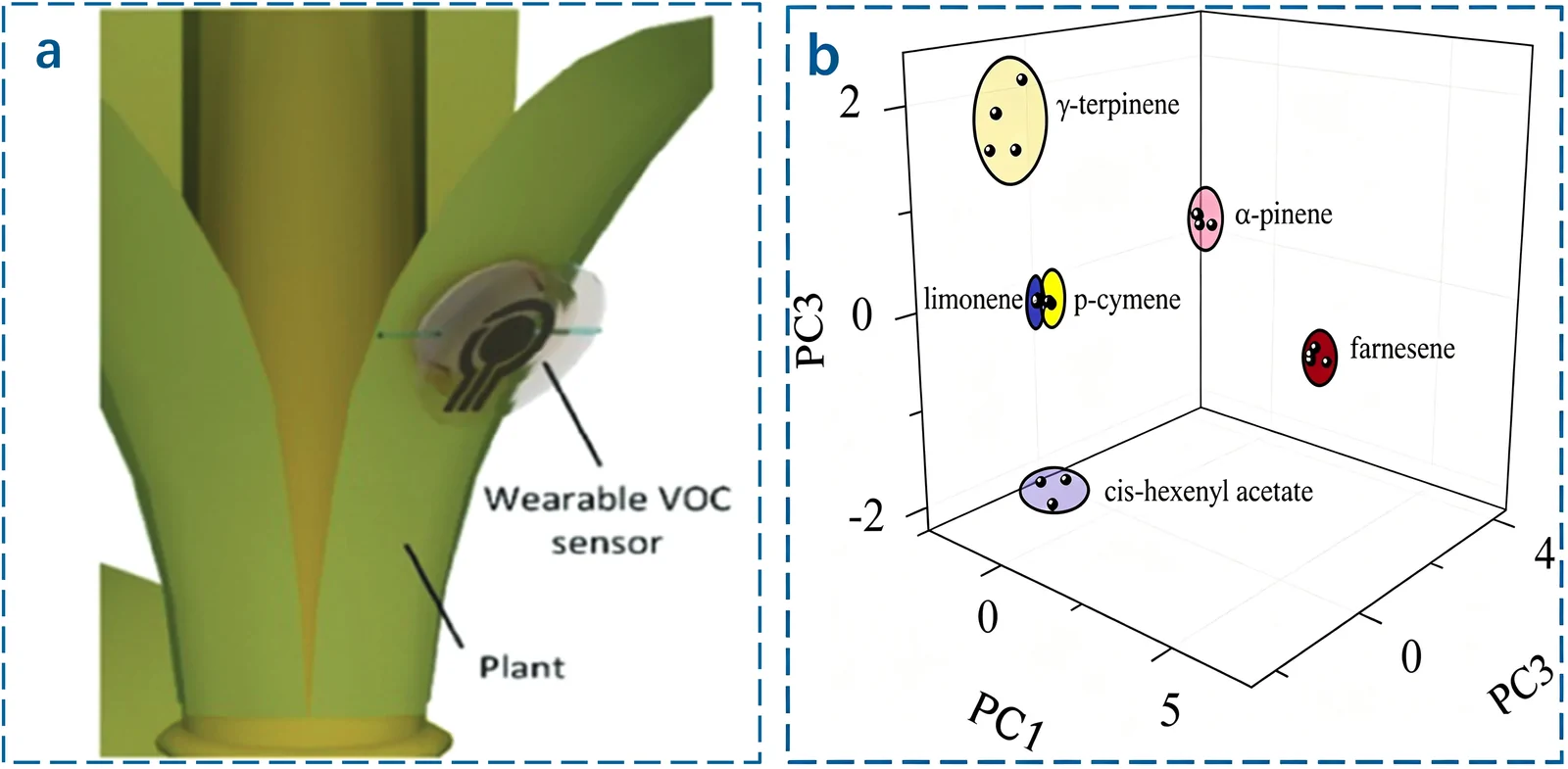
**(a)** Schematic diagram of a VOC sensor mounted on a leaf surface (Ibrahim et al., 2022); **(b)** Principal component plots of different plant chemical components (Weerakoon et al., 2012).

Under insect stress, plants release specific VOCs such as methanol, terpenes, and green leaf volatiles as defensive signals. Methanol is a major VOC emitted by plants. Methanol emissions reflect plants’ indirect defense against insects, facilitate intercellular communication, and enable plants to adapt to various environmental stresses. Both of the following sensors employ a design based on the time-current response mechanism but utilize slightly different materials. Their specificity for methanol detection has also been verified. However, the sensor data exhibit significant deviations from gold standard measurements (GC-MS), and the experimental setup has low relevance to real-world conditions. Bag and Lee (2021) attempted to measure methanol levels at different stages of maize development. However, Ibrahim et al. (2022) studied two maize genotypes. Neither study explored the response mechanism of methanol to abiotic or biotic stresses (**Figure 8a**). In contrast, Weerakoon et al. (2012) developed a sensor array based on various electroactive polymers to detect multiple phytochemicals (such as γ-terpinene, α-pinene, and limonene) released by plants in response to insect infestation. The array comprises carbon/polymer composite sensors, polystyrene sensors, and PANI sensors. By leveraging the differences in chemical structure and crystallinity among these polymers, the array achieves differentiated responses to various VOCs. Furthermore, principal component analysis was successfully employed to construct characteristic fingerprints for each compound, thereby effectively distinguishing between volatile signals induced by different insect pests (**Figure 8b**). This sensor array is low-cost, easy to microfabricate, and capable of operating at room temperature, making it suitable for field deployment and offering a new technical approach for early pest warning. Electronic nose technology has demonstrated excellent performance in detecting grain storage pests (such as rice weevils and grain beetles) and microbial contamination, with pattern recognition algorithms enhancing classification accuracy (Badgujar et al., 2026; Zheng et al., 2023a). These technologies provide effective tools for intelligent plant protection management in the field and in storage. The pest stress detection studies further reveal a critical gap between laboratory proof-of-concept and field-ready pest warning systems. The methanol sensors developed by Ibrahim et al. (2022) and Bag and Lee (2021) demonstrated sensitivity to methanol emissions under controlled greenhouse conditions but exhibited significant deviations from GC-MS gold standards, highlighting the challenges of achieving quantitative accuracy in complex field environments. Weerakoon et al. (2012) successfully demonstrated PCA-based discrimination of six insect-induced terpenoids using a polymer-based sensor array, yet the sensor response required 2 hours to reach equilibrium, far too slow for early pest warning applications where rapid detection is essential to initiate timely intervention. Moreover, the experimental setup involved exposing sensors to pure chemical standards in laboratory gas chambers rather than to actual insect-infested plants in field conditions, leaving important questions unanswered regarding sensor performance under realistic VOC mixtures, variable environmental conditions, and the presence of competing biological emissions. Electronic nose technology has shown success in grain storage pest detection (Badgujar et al., 2026; Zheng et al., 2023b), but the controlled conditions of storage environments differ substantially from open-field agricultural settings where wind dilution, diurnal emission variability, and background VOC interference dominate. These contrasts underscore the urgent need for field-validated pest detection studies that bridge the gap between laboratory demonstrations and practical agricultural deployment.

### 4.3 Assessment of growth status and vitality

As a gaseous plant hormone, ethylene regulates various processes such as fruit ripening, organ senescence, and stress responses and abrupt changes in its concentration often serve as an early sensitive indicator of stress (Wang et al., 2013; Zhang et al., 2024b; Binder, 2020; Wang et al., 2025; Zhang et al., 2024c). In recent years, driven by the demand for high sensitivity, high selectivity, flexible adaptability, and field applicability, researchers have made continuous breakthroughs in areas such as sensitive materials, sensing mechanisms, device structures, and fabrication processes, propelling wearable gas sensors from laboratory prototypes to practical applications in real-time plant monitoring. Before delving into the specific sensing strategies, it is important to clarify that the studies reviewed below differ substantially in their intended application scenarios. They can be broadly categorized into three groups: plant-surface wearable sensing, where sensors are directly attached to leaves or stems for *in situ*, long-term monitoring of living plants; fruit/package monitoring, where sensors are placed in storage containers or attached to fruit surfaces for postharvest quality control; and chip-based gas sensing, where sensors are fabricated on rigid substrates and operated in laboratory or industrial settings without conformal contact with plant tissues. While all these strategies contribute to ethylene detection, only those in the first category qualify as plant-wearable gas sensors in the strict sense. The following discussion explicitly distinguishes these categories to clarify the current status and future directions of truly wearable ethylene sensors for plant health monitoring.

**Figure 9:**
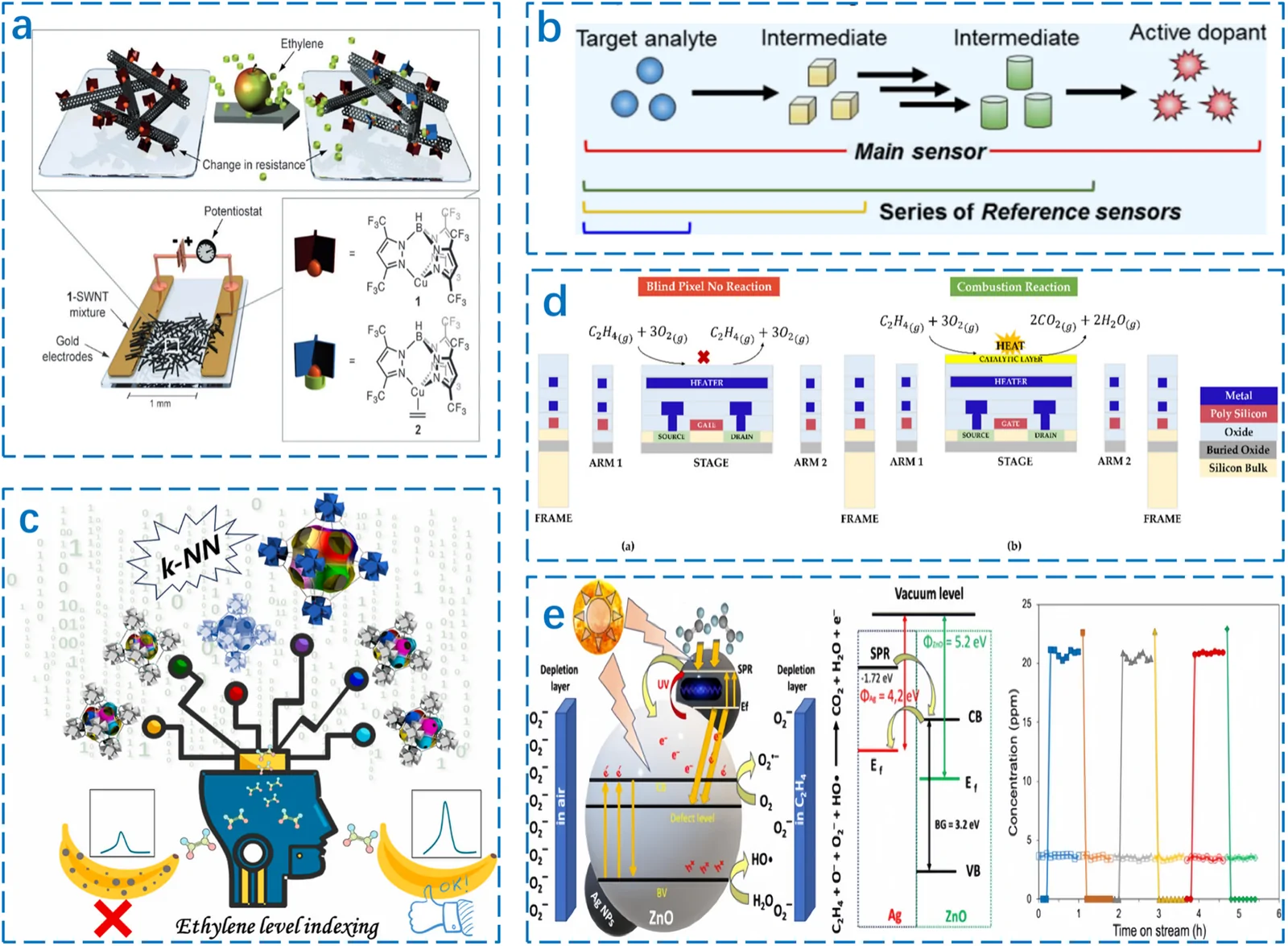
**(a)** Detection of ethylene using a chemical resistance sensor (Esser et al., 2012); **(b)** The target analyte is converted into an active dopant via CR and then detected by a chemical impedance sensor, the responses of the main sensor and the reference sensor are compared to extract the true response from the target analyte (Ishihara et al., 2020); **(c)** Schematic diagram of a MOF-based QCM electronic nose array for ethylene detection and fruit ripeness determination (Sun et al., 2024); **(d)** Schematic cross-sectional view of the GMOS sensor pixel structure. Blind pixel (without catalyst) (left), active pixel (covered with a catalyst layer) (right); both incorporate a heating resistor and a suspension (Ashkar et al., 2025); **(e)** Mechanism of interaction between the ZnO-Ag heterojunction and ethylene under visible light irradiation (Fernández-Ramos et al., 2025).

Regarding the mechanisms of ethylene recognition and sensing, early research primarily focused on direct sensing strategies. However, due to ethylene’s nonpolar nature and weak interactions, it has been difficult to achieve both high sensitivity and selectivity. Esser et al. (2012) composite copper(I) complexes and SWCNTs (**Figure 9a**). By leveraging the specific coordination interaction between copper and ethylene to modify the electrical properties of carbon nanotubes, this approach enables ethylene detection at the 0.5 ppm level, laying the foundation for carbon-based resistive ethylene sensing. This strategy falls into the fruit/package monitoring category, as the sensor was validated by measuring ethylene emissions from fruits enclosed in a gas flow chamber. However, this system exhibits significant responses to interferents such as ethanol and acetaldehyde, limiting its applicability in complex field environments. To address the issue of poor selectivity in direct sensing, Ishihara et al. (2020) proposed a cascade reaction strategy (Wacker oxidation + condensation to release acid) (**Figure 9b**), which converts ethylene into HCl that can be efficiently detected by SWCNTs. The system achieves a sensitivity of 10.9%/ppm and a detection limit as low as 0.2 ppm. Additionally, interference is eliminated through the use of a master-reference sensor array, enabling stable operation without the need for baseline correction. This significantly enhances detection reliability in complex atmospheres. This work represents a chip-based gas sensing approach, as the sensor was fabricated on rigid substrates and operated in a laboratory gas-flow system without conformal integration with plant surfaces. However, the system requires an operating temperature of 40°C, and there is still room for optimization in terms of flexibility and low-power consumption. Sun et al. (2024) constructed an electronic nose array by integrating six functionalized UiO series MOFs with a QCM (**Figure 9c**). By enhancing ethylene capture through π-π interactions and pore size matching, and combining this with machine learning, a detection limit of 0.27 ppm and a quantitative accuracy of 98.89% were achieved, enabling accurate discrimination of ripening stages in banana and kiwifruit. The integration of sensor arrays and intelligent algorithms improves the accuracy of growth status evaluation. This study is categorized as fruit/package monitoring, as the QCM-based sensor array was designed for ripeness indexing of detached fruits rather than on-plant wearable sensing. However, the flexibility and adhesion of QCM devices still require optimization to accommodate wearable applications. Nevertheless, the three aforementioned strategies are respectively limited by rigid substrates and reliance on external gas pathways, as well as issues such as high operating temperatures and low system integration, making it difficult to directly integrate them into portable or wearable gas sensing platforms. In the field of bioaffinity and visual recognition, researchers have taken a novel approach to developing biosensing strategies. Vong et al. (2019) developed an artificial metalloproteinase (ArM) fluorescent probe AEP, with albumin as the scaffold to embed a ruthenium catalyst. Fluorescence turn-on was achieved via ethylene-triggered cross-metathesis reaction, enabling direct spatiotemporal imaging of endogenous ethylene on fruits and Arabidopsis leaves. It exhibits excellent biocompatibility and causes no tissue damage. This fluorescence-based imaging approach is distinct from wearable sensing; while it provides valuable spatial information on ethylene distribution in plant tissues, it relies on fluorescence microscopy for signal readout and is not configured as a portable, continuous monitoring device. However, as this probe is a solution-based optical system, it relies on fluorescence microscopy for detection and lacks the real-time, portable, and wireless monitoring capabilities required for wearable devices. To complement this, Gal-Oz et al. (2023) developed a chitosan-molybdenum bio-composite electrochemical chip that utilizes the Mo^6+^/Mo^5+^ redox cycle to achieve a specific response to ethylene. The material is flexible and film-formable, and the miniaturized chip is easily integrated, providing a material foundation for wearable electrochemical sensing units. This work is best described as a chip-based sensing platform; although the biocomposite material exhibits flexibility, the device was not configured as a wearable patch for plant attachment, and its response speed remains insufficient for real-time stress monitoring. Unfortunately, the development of a flexible substrate and a wearable structure was not completed, and the response speed is relatively slow, making it difficult to meet the requirements for real-time monitoring of early-stage stress.

In the field of micro-electro-mechanical systems and low-power integration, Ashkar et al. (2025) fabricated a GMOS micro catalytic sensor based on CMOS-MEMS technology (**Figure 9d**). By leveraging the synergistic effect of Pd-Pt bimetallic catalyst, this approach reduces the activation energy and operating temperature required for ethylene combustion, enabling detection across a wide range of 5–1000 ppm. The power consumption was reduced by 23.41% compared to single-metal catalysts, exhibiting the advantages of miniaturization, low power consumption and high stability. This sensor is a chip-based gas sensing device fabricated on a rigid CMOS-MEMS platform, designed for industrial and agricultural storage applications rather than direct attachment to plant surfaces. The miniaturized architecture provides hardware support for on-chip integration and long-term on-chip monitoring, demonstrating the potential for future integration into portable sensing systems. However, the rigid substrate and operating temperature requirements still present challenges for direct adaptation to plant-wearable formats, addressing the shortcomings of traditional catalytic sensors such as high operating temperatures, high power consumption, and lack of wearability.

In terms of flexibility and room-temperature operation, Fernández-Ramos et al. (2025) developed a flexible chemical resistance-based ethylene sensor using ZnO-Ag nanocomposites, with PET-ITO as the flexible substrate and PVDF as the film-forming matrix, and EMIMZ ionic liquid as a stabilizing additive. By regulating the morphology of ZnO (nanospheres, nanorods) and combining it with Ag and GO, they constructed a flexible sensing unit capable of adhering to the surface of fruits (**Figure 9e**). The sensor operates at room temperature (25°C), exhibits a selectivity for ethylene of up to 100%, and has a detection limit of 0.25 ppm. With response and recovery times of 35–40 s and 110–120 s, respectively, it has successfully enabled dynamic monitoring of the banana ripening process, representing the most relevant example of flexible, attachable ethylene sensing among the strategies discussed here. Although this work focuses on fruit surface attachment for postharvest monitoring rather than on-leaf plant health assessment, its flexible substrate, room-temperature operation, and low power consumption make it a promising prototype for future adaptation to plant-wearable gas sensing systems. This demonstrates its practical potential as a low-cost, low-power, flexible, wearable ethylene sensor, and provides significant support for the practical application of wearable gas sensors in the field of *in vivo* plant monitoring. The ethylene sensing strategies reviewed in this section illustrate a spectrum of technological readiness, ranging from laboratory-based chip sensors to field-deployable flexible devices. Esser et al. (2012) and Ishihara et al. (2020) demonstrated impressive ethylene sensitivity (0.2-0.5 ppm) using carbon nanotube-based chemiresistive and cascade-reaction approaches, but these sensors were validated on rigid substrates under laboratory gas-flow conditions, with limited relevance to plant-surface wearable applications. Sun et al. (2024) achieved 0.27 ppm LOD with MOF-based QCM arrays, yet the QCM platform’s rigidity and reliance on frequency measurement instrumentation currently preclude wearable formats. Vong et al. (2019) and Gal-Oz et al. (2023) introduced innovative optical and electrochemical detection strategies, but their solution-phase operation and chip-based architectures respectively restrict continuous, on-plant monitoring. In contrast, the ZnO-Ag flexible sensor developed by Fernández-Ramos et al. (2025) represents a significant step toward practical wearable ethylene sensing, offering room-temperature operation (25°C), ~100% selectivity for ethylene, and a LOD of 0.25 ppm, comparable to or better than many laboratory-based sensors, while maintaining flexibility and low power consumption. However, even this flexible sensor was validated primarily on fruit surfaces under laboratory conditions rather than on living plant leaves in field environments. The sensor demonstrated good repeatability (99.0%) and long-term stability (90 days), yet its response saturated at high ethylene concentrations, limiting its dynamic range for practical monitoring scenarios where ethylene levels vary widely. Ashkar et al. (2025) addressed the challenge of low-power operation through CMOS-MEMS integration with Pd-Pt bimetallic catalysts, achieving 23.41% power reduction compared to conventional catalytic sensors. Nevertheless, the rigid substrate and chip-based architecture of the GMOS device limit its wearable applicability. Collectively, these studies reveal a clear trajectory: while laboratory-based ethylene sensors have achieved remarkable sensitivity and selectivity, translating these capabilities into field-deployable, plant-wearable devices requires further advances in flexible substrate engineering, power management, and real-world validation under the variable conditions encountered in agricultural settings.

To systematically review the progress in the application of wearable gas sensors in plant health monitoring, this paper categorizes and summarizes the research across three major scenarios: abiotic stress, biotic stress, and growth status assessment, covering monitoring targets, characteristic VOCs, sensor types, key performance, and representative literature, as detailed in **Table 2**. In summary, current research has established a technical system featuring parallel mechanisms (resistive, electrochemical, optical, and mass-sensitive), synergistic materials (carbon-based, biocomposite, MOF, and catalytic metals), and supporting processes (flexible printing, array recognition, and MEMS integration). Core challenges have been gradually addressed, including low sensitivity, poor selectivity, insufficient flexibility, weak environmental anti-interference, and difficulty in *in vivo* monitoring. Wearable gas sensors have evolved from single-performance optimization to precise, real-time, noninvasive assessment of plant growth, ripening, and stress vigor, providing novel portable monitoring tools for smart agriculture and plant physiology research.

**Table 2:** Typical applications of wearable gas sensors in plant health monitoring.

VOCs	Sensor active layer	Substrate/encapsulation materials	Build method	Sensor mechanism	LOD	Linear range	Sensitivity	Response/recovery time	Selectivity	Humidity tolerance	Bending stability	Field validation	Applications	Transmission method	Power supply method	References
Methanol	PtNPs/poly(ATD)	PET	Screen printing	Amperometric	0.5 ppm	0.5–500 ppm	1.14 nA/ppm	<10 s/-	Good vs. ethanol, isopropanol, acetone, acetic acid, acetaldehyde	Stable at 5–100% RH	–	Yes (field, maize)	Detection of maize pest stress	Wired transmission	Wired power supply	Ibrahim et al. (2022)
Methanol	PtNPs/ZnONRs@ZIF-8	PET + carbon conductive ink	Screen printing + dip coating + spray coating	Dual-channel chemical resistor	133 ppb	3.5–100 ppm	-24.37 Ω/log(ppm)	-/~300 s (regeneration)	Good vs. CH_4_, NH_3_, C_2_H_4_	40–90% RH	–	Yes (cotton, greenhouse, 23 days)	Detection of nitrogen deficiency in cotton	Wired transmission	Wired power supply	Sangmen et al. (2026)
α-Pinene	MIP (MAA/EGDMA/AIBN)	Kapton + Ag conductive ink	Screen printing + drop-casting + spin coating + UV curing	Chemiresistive (MIP-based)	62.15 ppb	600–1000 ppb	0.0006 Ω/ppb	–	Good vs. CH_3_OH, CH_4_, NH_3_, C_2_H_4_	40–90% RH	–	Yes (cotton, greenhouse, 23 days)	Nutrient stress (N deficiency)	Wired transmission	Wired power supply	Sangmen et al. (2026)
α-Pinene	PEVA/MWCNT	PET + Au IDEs	Printed electrodes + dip-coating	Chemiresistive	0.5 ppm	0.5–40 ppm	8.32 kΩ/ppm	-/8 s (95% recovery)	Good vs. 7 plant VOCs	Stable at 40–90% RH	Stable under PET bending	Yes (Platycladus orientalis, mechanical damage + drought)	Detection of mechanical damage and drought stress in Thuja orientalis	NFC	Passive (NFC)	Zheng et al., (2023b)
Ethylene	ZnO-Ag (0.6 mM)	PET-ITO	Electrodeposition	Chemiresistive	30 ppm	30–100 ppm	–	4–6 min/10–15 min	Good vs. ethanol, methanol, acetone, xylene, toluene	Response increases with RH (50–80%)	–	No (laboratory only)	Fruit salt stress detection	Wired transmission	Wired power supply	Sholehah et al. (2021)
Ethylene	SWCNTs-PdNPs-PMs	Paper-based + Au IDEs	Laser direct writing technology	Chemiresistive	100 ppb	0.1–1 ppm; 10–100 ppm	29.68 (0.1–1 ppm); 0.41 (10–100 ppm) (%/ppm)	~6 s/~3 s	–	Stable at <70% RH; decreases at >70% RH	Good (1200 cycles, <0.36% change)	Laboratory fruit test (banana, 12 days)	Detection of salt stress in bananas and other fruits	Wired transmission	Battery-powered	Yan et al. (2023)
Green leaf volatiles such as (E)-2-hexenal	rGO + thiourea; AuNP@rGO with ligands (ITP, BTP, CTP, FTP, NTP, MTP)	PI film + kirigami structure + AgNW electrodes	Laser cutting + stencil dip coating + spin coating	Chemiresistive	0.13–3.9 ppm	10–50 ppm	VOC-dependent	<20 s/~60 s	>97% classification accuracy (13 VOCs)	Calibratable (signal decreases at high RH)	Excellent (< ± 0.2% at 50% strain	Yes (tomato leaf, late blight + mechanical damage)	Late blight stress in tomatoes	Wired connection	Wired power supply	Li Z. et al. (2021)
Plant VOCs such as (E) -2-hexenal	Cys-functionalized Au NPs/NRs	Nitrocellulose films/cover slips and O- rings	Drip-on	Colorimetric method	0.4 ppm ((E)-2-hexenal);0.9-5.2 ppm (others)	0.1–100 ppm	VOC-dependent	<1 min/-	>95% blind test accuracy	Good (environmentally robust)	–	Yes (field-collected tomato leaves, 97.5% accuracy)	Non-invasive early diagnosis of late blight in tomatoes	Offline image capture	Battery- powered	Li Z. et al. (2019)
Plant VOCs such as α- Pinene	Carbon/polymer composites (PE, PEVA, etc.)	Monocrystalline silicon + Au IDEs	deposition	Chemiresistive Array	100 ppm	–	–	<2 h/-	PCA-based discrimination of 6 VOCs	Stable under different humidity	–	No (laboratory only)	Early detection of pests in various agricultural crops	Wired transmission	Wired power supply	Weerakoon et al. (2012)
NH_3_	PANI + CoTPP	ITO-PET	Electrodeposition	Chemiresistive	0.83 ppm	50–500 ppm	0.18 (%/ppm)	55 s/269 s	Good vs. N_2_, O_2_, CO_2_, H_2_, C_2_H_4_	Response increases with RH (20–90%)	>90% after 500 cycles (5mm radius)	No (laboratory only)	Environmental gas monitoring	Wired transmission	Wired power supply	Cai et al. (2023)
Hexanal/Acetone	Au@AgNWs + MWCNTs + FTP/CTP/BTP/ITP ligands	PDMS + hydrophobic sol-gel layer (MTMS/TMOS)	Spray coating + Masking + Transfer printing + Selective dipping	Chemiresistive	100–500 ppm	100–500 ppm	FTP/CTP highest (ligand-dependent)	<2min/<2 min	Ligand-dependent (halogen bonding)	Excellent (90% RH with sol-gel protection)	–	Yes (tomato, drought, salt, TSWV, early blight)	Plant stress (abiotic + biotic)	Wired transmission	Wired power supply	Lee et al. (2023)
NO_2_	MWCNTs	PDMS + amphibian toe pad-mimicking adhesive layer	Biomimetic Molding + Electrochemical Deposition + Transfer Molding	Chemiresistive	1.39 ppb	7.2 ppb – 50 ppm	0.01134 (%/ppb)	–	Excellent vs. NH_3_, ethanol, acetone, CO_2_, CO	Response increases with RH (10–80%)	Stable under both convex and concave bending	Yes (coffee leaf, automotive exhaust NO_2_)	NO_2_ pollution monitoring in coffee trees	Wired transmission	Wired power supply	Kim et al. (2026)
VOCs from green leaves such as (E)-2-hexenal	Dye/UiO-66(Zr)@PDMS (DUP)	Flexible PDMS	Extrusion printing	Colorimetric method	0.5–1 ppm (LOR)	0.5–25 ppm	Concentration-dependent	~20 min/-	PCA-based discrimination of 8 VOCs	Excellent (stable at 90% RH)	Excellent (strain up to 50%)	Yes (wheat leaf, scab detection, 2 days post-inoculation)	*In-situ* detection of wheat fusarium head blight	The scanner captures the image	No power required	Wang et al., (2024b)
Ethylene	PdNPs-SWCNTs@Cu-MOF-74 (SPM)	PET + PI solution + Au IDEs	Laser direct writing technology	Chemiresistive	31 ppb	0.1–1 ppm; 1–10 ppm; 10–100 ppm	16.41 (0.1–1 ppm); 1.11 (1–10 ppm); 0.1 (10–100 ppm) (%/ppm)	200 s/50s	Excellent vs. ETOH, H_2_S, NO_2_, NH_3_, CO	Stable at 40–70% RH; decreases at >70% RH	–	No (laboratory fruit test: kiwi)	Detection of salt stress in kiwifruit and other fruits	Wired transmission	Wired power supply	Yan et al. (2024)
(E)-2-hexenal	AIE-Au nanoclusters + cysteine	Agarose hydrogel	Mold casting	Fluorescence method	0.61 ppm	1–70 ppm (hydrogel); 0.1–150 ppm (solution)	Concentration-dependent	~1h/-	Good vs. other VOCs (cross-reactivity with 2-ethyl-2-hexenal)	Good (hydrogel microenvironment)	–	Detached leaf test (potato, late blight, 2 days)	Early detection of late blight in potatoes	Offline image capture	Battery- powered	Gan and Wang (2024)
Ethylene	ZnO-Ag (1 wt%) nanorods (HM3)/ZnO-Ag-GO (HM5)	PET-ITO + Ag electrodes	Spin-coating method	Chemiresistive	0.25–0.26 ppm	0–10 ppm	1.80 (HM3)/1.70 (HM5) (%/ppm)	35–40 s/110–120 s	~100% selectivity for ethylene	Stable at 10–100% RH	–	No (laboratory fruit test: banana, 20 days)	Real-time monitoring of banana ripeness	Wired transmission	Wired power supply	Fernández-Ramos et al. (2025)

## 5 Challenges and future prospects for wearable gas sensors

Despite the substantial progress reviewed in the preceding sections, the commercialization of wearable gas sensors for plant health monitoring remains in its early stages, with several significant barriers to widespread agricultural adoption. These challenges can be broadly categorized into technical, economic, and regulatory domains.

From a technical perspective, the trade-off between sensitivity and selectivity remains a fundamental challenge: sensors with high sensitivity often respond to multiple VOCs and environmental interferents, while sensors with high selectivity typically rely on specialized recognition elements (such as molecularly imprinted polymers, specific catalysts) that may compromise sensitivity or long-term stability. Standardization of sensor performance metrics, including sensitivity, selectivity, response time, recovery time, and stability, is currently lacking across the literature, making meaningful comparisons between different sensor technologies difficult and hindering technology selection for specific agricultural applications. Furthermore, the scalability of sensor fabrication from laboratory-scale prototypes to cost-effective mass production remains unresolved: many reported sensors rely on manual fabrication processes (such as drop-casting, spin-coating, electrodeposition) that are not readily transferable to high-throughput manufacturing. The integration of wireless communication, power management, and data processing electronics into compact, lightweight sensor packages also presents substantial engineering challenges that have been addressed in only a handful of studies (Zheng et al., 2023a; Lee et al., 2023).

Economically, the cost-effectiveness of wearable VOC sensors for agricultural applicationsremains uncertain. While individual sensor fabrication costs have been estimated at <$1 per unit in some studies (Li Z. et al., 2021), the total system cost, including wireless transceivers, power sources (batteries or energy harvesters), data acquisition electronics, and data analytics platforms, is substantially higher, potentially exceeding the economic threshold for widespread adoption in low-margin agricultural systems. The limited lifespan of current sensors (typically days to weeks) due to biofouling, baseline drift, and material degradation further increases the total cost of ownership through frequent replacement and recalibration.

Regulatory and user adoption barriers also impede commercialization. Standardized testing protocols for evaluating plant-wearable sensors under field conditions are currently absent, making it difficult for end-users to assess and compare sensor performance. Data ownership, privacy, and integration with existing farm management systems remain largely unaddressed. Moreover, the agriculture sector typically operates with conservative adoption of new technologies, requiring robust demonstration of economic return on investment, a metric that has not yet been established for plant-wearable VOC sensors.

Addressing these commercialization challenges will require interdisciplinary collaboration among materials scientists, engineers, agricultural researchers, and industry stakeholders to develop standardized testing frameworks, scalable fabrication processes, and economically viable sensor systems that deliver tangible value to farmers and agricultural producers.

Although wearable gas sensors have made significant strides in the field of plant health monitoring, their transition from the laboratory to large-scale field applications still faces multiple challenges.

(1) The quantitative relationship between VOCs release profiles and plant health status requires further investigation. Currently, most studies remain at the qualitative level, capable only of determining overall changes in the intensity of stress signals. Establishing precise dose-response models linking stress levels (such as pest density and damage severity) to the release rates or concentrations of specific VOCs is a prerequisite for quantitative diagnosis. However, field-based VOC monitoring introduces additional physical complexities that are absent in controlled laboratory settings: wind-driven convective dilution can reduce local VOC concentrations by orders of magnitude, while water films on leaf surfaces, formed by dew, rain, or high humidity, can severely impede gas diffusion from the stomatal or cuticular emission sites to the sensor interface. These environmental factors not only attenuate signal strength but also introduce temporal variability that complicates the correlation between sensor readings and actual stress intensity. In the future, sensors must be rigorously calibrated and validated against standard methods such as GC-MS. A standardized VOCs “fingerprint” database should be established, and through large-scale field experiments and cross-validation with GC-MS, precise dose-response models linking stress levels to VOCs dynamics should be constructed, thereby achieving a leap from signal changes to physiological diagnosis. The root cause of the aforementioned issues lies in the fact that there is no absolute ‘one-to-one’ correspondence between a single VOC marker and a specific type of stress. VOC release by plants is a complex phenotype resulting from the combined effects of multiple physiological and ecological factors; it is regulated by a variety of factors, including species differences, tissue and organ types, developmental stages, circadian rhythms, temperature and humidity, and combined stresses. Consequently, future research must move beyond the one-dimensional approach of ‘identifying a specific VOC as a probe for a particular stress’ and instead focus on constructing a VOC fingerprint database covering multiple species, stress types and environmental contexts. By utilizing sensor arrays and machine learning algorithms to achieve precise identification of stress types, this approach represents not only a research topic in basic plant physiology but also a necessary prerequisite for the transition of wearable gas sensors from the laboratory to field applications.

(2) The overall performance of sensors still needs to be improved in two dimensions: environmental adaptability and molecular recognition specificity. At present, environmental disturbances, especially drastic fluctuations in humidity and temperature, significantly restrict the long-term operational stability and gas recognition selectivity of sensors, leading to frequent issues such as sensing signal drift, unstable baseline, and cross-response interference. Beyond these atmospheric factors, long-term field deployment introduces additional failure modes. Biofouling, the accumulation of microorganisms, plant exudates, and airborne particulates on sensor surfaces, can progressively degrade sensitivity and response kinetics over extended deployment periods. Leaf growth and surface expansion can cause mechanical detachment or delamination of the sensor from the plant tissue, compromising conformal contact and signal fidelity. Rain exposure not only introduces physical erosion but also creates transient water films that interfere with gas diffusion and sensor–leaf interfacial adhesion. UV radiation accelerates the photodegradation of polymeric substrate materials such as PDMS and PET through chain scission and crosslinking reactions, leading to embrittlement, cracking, and eventual device failure (Shen et al., 2026). Seasonal variations in temperature, humidity, and foliar phenology further introduce baseline drift and dynamic range shifts that complicate long-term data interpretation. Collectively, these factors necessitate the development of self-cleaning or anti-biofouling surface coatings, UV-stable encapsulation layers, and stretchable sensor designs that accommodate leaf expansion, as well as the establishment of systematic replacement schedules tailored to specific deployment environments. Therefore, future research should focus on the development of sensitive materials and novel packaging protection strategies resistant to temperature and humidity interference, as well as the practical considerations of field longevity and maintainability. By constructing biomimetic interfaces, applying molecular imprinting techniques, and adopting multi-sensor array pattern recognition algorithms, precise identification of target gases and collaborative discrimination of multiple markers in complex plant volatile systems can be achieved. In addition, to meet the strategic needs of green agriculture and sustainable development, the introduction of degradable flexible electronic materials is crucial. It is recommended to prioritize natural polymer materials such as cellulose-based and lignin-based substrates as sensing platforms or encapsulation layers to enable zero-waste deployment.

(3) The conflict between power consumption and power supply remains critical: the energy output and storage efficiency of self-powered systems are insufficient to support long-term, high-density data acquisition. At present, most devices still rely on traditional chemical batteries or external power sources, which not only limits the endurance of sensors but also significantly restricts their potential for long-term *in-situ* deployment and large-scale application in field environments. Although self-powered strategies based on triboelectric nanogenerators, solar energy, contact electrification, and other mechanisms have shown promising application prospects, their core performance indicators such as output power density, energy storage efficiency, and flexible integration compatibility, still cannot meet the stringent requirements of wearable sensors for stability and continuity. Future research should focus on the collaborative optimization of ultra-low-power system design and multi-source energy harvesting technologies. Examples include the development of miniaturized high-efficiency electronic components, the design of ultra-low-power circuit architectures for flexible electronics, and the integrated innovation of multi-mode energy harvesting systems. The goal is to establish a self-powered, long-endurance, flexible sensing power supply system that completely breaks through energy limitations, providing core technical support for long-term *in-situ* monitoring of plant health.

(4) System integration and intellectualization. Faced with complex and variable field ecological environments, the single-variable sensing mode can hardly meet the diversified demands for precise plant health monitoring. Moreover, the physical act of attaching sensors to living plant tissues introduces physiological perturbations that are often overlooked: sensors can shade the leaf surface, reducing photosynthetic activity, while mechanical pressure from the device may induce localized stress responses that alter the very VOC emissions being measured. Continuous leaf growth can cause gradual delamination, progressively degrading the sensor–leaf interfacial contact over time (Li et al., 2024). Long-term baseline drift, driven by gradual changes in sensor material properties and environmental conditions, further compounds the challenge of data interpretation. Cross-sensitivity among multiple co-emitted VOCs, particularly between structurally similar terpenes or between alcohols and aldehydes, remains a persistent challenge for single-channel sensors, even with optimized sensitive materials. Multifunctional and multivariable integration has become the core development direction for constructing the next-generation wearable gas sensor system. The key to overcoming these field-specific challenges lies in moving beyond single-analyte detection toward integrated sensor arrays, coupled with *in-situ* calibration protocols and machine learning algorithms that can decouple mixed signals and compensate for environmental and physiological interferences. Through the collaborative design of cross-responsive sensitive materials and independent multi-channel signal readout, multimodal and multivariable sensing systems can effectively attenuate environmental interference, and significantly improve the molecular recognition selectivity toward target gases and the stability against long-term signal drift. Furthermore, the in-depth system integration of multi-source fusion sensing platforms aims to break the limitations of traditional single-index monitoring. This platform organically combines gas component monitoring (such as VOCs) with the acquisition of biological indicators including leaf temperature, relative humidity, ion flux, and photosynthetic physiological parameters. Relying on deep learning-driven multidimensional feature extraction and intelligent pattern recognition algorithms, it realizes the precise decoupling, dynamic evaluation, and real-time intelligent decision-making of plant stress signals, thereby providing more comprehensive and reliable health diagnosis evidence for smart agriculture.

(5) Wireless transmission methods need to transition from traditional wireless technologies (such as Bluetooth, Wi-Fi, and ZigBee) to IoT-specific transmission methods. Bluetooth and Wi-Fi have limited transmission ranges and cannot cover large-scale farmland areas. Furthermore, their high power consumption creates a significant conflict with the limited battery life of wearable sensors. Although ZigBee supports multi-node networking, its signals are easily obstructed by obstacles such as crop canopies and uneven terrain, resulting in poor transmission stability and frequent issues such as data loss and delays. In the future, the development of data transmission technology will center on the practical needs of field applications, focusing on core objectives such as low power consumption, long-range transmission, high stability, and multi-node coordination. This will drive the deep integration of low-power wide-area networks (LPWAN), the internet of things (IoT), and edge computing technologies, optimize transmission architectures, and enable the long-range, low-power, synchronous transmission of multi-sensor data while reducing signal interference caused by complex field environments.

In summary, the widespread adoption of wearable gas sensors hinges on the development of a closed-loop intelligent system linking plants, sensors, and the cloud. With the convergence of materials science, nanotechnology, the IoT, artificial intelligence, and energy harvesting technologies, wearable gas sensors are poised to evolve into highly autonomous, intelligent, and long-lasting plant health monitoring platforms. These platforms will become a core component of smart agriculture infrastructure, driving a paradigm shift in crop health management. These platforms will facilitate a leap from single-point monitoring to crop population phenomics. By establishing unified sensor performance evaluation standards and data-sharing platforms, they will accelerate the transition from prototype validation to industrial implementation, providing robust technological support for precision agriculture, climate-resilient agriculture, and sustainable development. Ultimately, this technology will not only enhance crop yields and quality but also make a significant contribution to global food security and ecological conservation.

## 6 Summary

As an emerging technology for the *in-situ* monitoring of plant health, wearable gas sensors exhibit irreplaceable advantages in the precise detection of plant VOCs, owing to their superior flexibility, real-time *in-situ* capability, and non-destructive nature. This paper systematically reviews the structural design, sensing mechanisms, and fabrication technologies of such sensors, as well as their typical applications in three major fields: abiotic stress monitoring (drought, salt stress, and nutrient deficiency), early diagnosis of pest and disease stress, and evaluation of plant growth status and vigor. A review of representative research advances in recent years reveals that the technology is undergoing a systematic transition from quantitative detection of single parameters to multimodal integrated sensing, collaborative recognition of multiple biomarkers, and intelligent data analysis. Specifically, the cross-integration of chemiresistive, electrochemical, optical, and other sensing mechanisms, coupled with wireless communication technologies such as the IoT and deep integration with machine learning algorithms, enables sensors to sensitively capture and selectively identify key biomarkers including α-pinene, ethylene, (E)-2-hexenal, and methanol in complex field environments, preliminarily realizing intelligent classification and early warning of stress types. Furthermore, the introduction of self-powered strategies based on triboelectric nanogenerators (TENGs), solar energy harvesting, and the application of flexible and biodegradable bio-substrates have effectively addressed challenges related to service life and eco-friendliness, while significantly improving the practicality and sustainability of long-term *in-situ* deployment in farmlands, laying a solid foundation for the application of wearable gas sensor technology in precision agriculture.

In summary, wearable gas sensors are not only tools for plant health monitoring, but a bridge connecting plant physiology and digital agriculture. Their ultimate value lies in enabling farmers to implement on-demand irrigation, fertilization, and pesticide application through continuous and accurate *in-situ* data feedback, thereby significantly reducing resource input, alleviating environmental pollution, and improving crop yield and quality. Against the backdrop of increasingly severe climate change, the advancement of this technology will provide strong technical support for safeguarding global food security and promoting the transformation of sustainable agriculture. We are confident that, with the deep integration of materials innovation, artificial intelligence, and the IoT, wearable plant sensing systems will become routine field equipment within the next 5–10 years, laying a solid foundation for the construction of an intelligent agricultural ecosystem with integrated sensing-decision-execution.
